# Dengue virus envelope epitope-specific antibodies are associated with protection from severe dengue

**DOI:** 10.1038/s44321-026-00485-7

**Published:** 2026-08-06

**Authors:** Sokchea Lay, Heidi Auerswald, Sievleang Heng, Sopheak Sorn, Sreymom Ken, Veronica Duran, David Esteban Rebellón Sanchez, Daniela Vinueza, Luis Angel Villar Centeno, Fernando Rosso, Shirit Einav, Anavaj Sakuntabhai, Felix A Rey, Kevin K Ariën, Sowath Ly, Veasna Duong, Giovanna Barba-Spaeth, Tineke Cantaert

**Affiliations:** 1 Immunology Unit, Institut Pasteur du Cambodge, Pasteur Network, Phnom Penh, Cambodia; 2 Virology Unit, Institut Pasteur du Cambodge, Pasteur Network, Phnom Penh, Cambodia; 3 Epidemiology and Public Health Unit, Institut Pasteur du Cambodge, Pasteur Network, Phnom Penh, Cambodia; 4 Department of Medicine, Department of Microbiology and Immunology, Stanford University, Stanford, CA USA; 5 Chan Zuckerberg Biohub, San Francisco, CA USA; 6 Clinical Research Center, Fundación Valle del Lili, Cali, Colombia; 7 Center for Care and Diagnosis of Infectious Diseases (CDI/INFOVIDA Foundation), Bucaramanga, Colombia; 8 Unit of Ecology and Emergence of arthropod-borne pathogens, Institut Pasteur, Université Paris Cité, CNRS UMR 2000, 75015 Paris, France; 9 Structural Virology Unit, Institut Pasteur, Université Paris Cité, CNRS UMR3569, 75015 Paris, France; 10 Virology Unit, Department of Biomedical Sciences, Institute of Tropical Medicine, Antwerp, Belgium; 11 Department of Biomedical Sciences, University of Antwerp, Antwerp, Belgium; 12 Structural Virology Unit, FlavImmunity Group, Université Paris Cité, Institut Pasteur, Paris, France; 13 Present Address: Virology Unit, Istituto Zooprofilattico Sperimentale dell’Abruzzo e del Molise, Teramo, Italy

**Keywords:** Immunology, Microbiology, Virology & Host Pathogen Interaction

## Abstract

Our understanding of a protective humoral immune response to dengue virus (DENV) remains limited. The envelope (E) protein is the main antibody (Ab) target. While anti-fusion loop (FL) epitope monoclonal Abs (mAbs) can induce antibody dependent enhancement (ADE) in vitro, some mAbs targeting quaternary epitopes can cross-neutralize different DENV serotypes. However, the contribution of each Ab subset to disease outcome remains poorly characterized. We defined DENV2 E epitope-specific Abs dynamics and assessed their association with disease outcome in a cohort of hospitalized and subclinical dengue patients during post-primary DENV2 infection. We quantified and isolated anti-E epitope-specific Abs and tested their enhancing and neutralizing capacity. During the critical phase, FL-targeting antibodies were increased in hospitalized patients compared to subclinical cases. Antibodies targeting quaternary epitopes were reduced in severe dengue compared to classical dengue fever patients. Functionally, quaternary epitope-targeting antibodies showed stronger neutralization and cross-neutralization properties, while FL-binding antibodies displayed stronger in vitro enhancement. These findings show that anti-DENV2 E epitope-specific Ab proportions correlate with disease susceptibility and severity, with important implications for novel vaccine design.

## Synopsis

Antibodies against dengue virus (DENV) can play both a protective and a disease enhancing role via antibody-dependent enhancement. The envelope (E) protein of the virus is the main target of the antibody (Ab) response. The composition, kinetics, and function of antibodies targeting different epitopes on the E protein, and their direct link to disease severity, remain insufficiently defined during natural infection.

- The proportion of fusion loop-targeting antibodies, known to be poorly neutralizing and infection enhancing, is increased in hospitalized patients compared to subclinical cases during the critical phase of infection.
- Antibodies targeting quaternary epitopes on the E-dimer interface are reduced in severe dengue patients compared to classical dengue fever patients.
- Functionally, isolated antibodies from plasma targeting quaternary epitopes showed stronger neutralization and cross-neutralization, while antibodies binding to the fusion loop displayed a stronger enhancing potential in in vitro assays.

## Abstract (web summary)

Antibodies against dengue virus (DENV) can play both a protective and a disease enhancing role via antibody-dependent enhancement. The envelope (E) protein of the virus is the main target of the antibody (Ab) response. The composition, kinetics, and function of antibodies targeting different epitopes on the E protein, and their direct link to disease severity, remain insufficiently defined during natural infection.

## Graphical abstract

**Figure:**
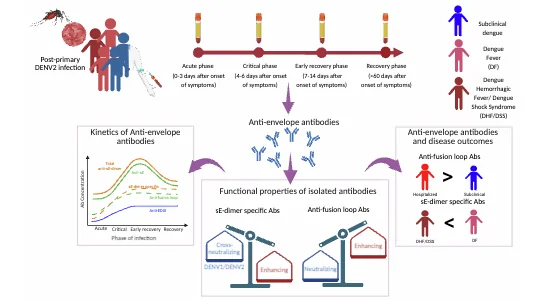

**Figure:**
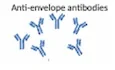

The paper explained **Problem**

There are currently no widely accepted and implemented correlates of protection for dengue. Neutralizing antibodies to the envelope (E) protein have been linked to protection in some studies. Monoclonal antibodies targeting the fusion loop are often weakly neutralizing and can mediate antibody-dependent enhancement (ADE) in vitro and in animal models, while some monoclonal antibodies targeting the E-dimer interface show serotype cross-neutralization. However, the composition, kinetics, and function of epitope-specific antibody subsets during natural secondary infection, and their direct link to disease severity in humans, remained insufficiently defined.

**Results obtained**

In a Cambodian cohort of pediatric cases with disease outcomes ranging from severe dengue to subclinical infection, we examined the dynamics of dengue E protein epitope-specific antibodies (Abs) during post-primary DENV infection. We found that the proportion of fusion loop-targeting Abs is increased in hospitalized patients compared to subclinical cases during the critical phase of infection, whereas the proportion of sE dimer-specific Abs is reduced in severe dengue patients compared to patients with classical dengue fever. Functionally, sE dimer-specific Abs showed stronger neutralization and cross-neutralization properties, while FL-targeting Abs were more enhancing in vitro. To our knowledge, this is the first direct evidence in humans linking the composition of anti-dengue E protein Ab subsets to both disease severity and functional capacity during acute infection. By extending prior work across distinct epidemiological settings and validating results with patient-derived Ab fractions, our study highlights the potential of epitope-specific Ab profiles as correlates of protection, predictors of severe disease, and provides insight into targets of vaccine design.

**Clinical impact**

Taken together, current evidence indicates that not all dengue virus-specific Abs are equal: their epitope specificity, functional profile, and kinetics critically influence whether they mediate protection or contribute to disease. Our findings add direct evidence from natural human infections showing that the relative balance of epitope-specific Abs during the critical phase correlates with disease outcome. This implies that protective immunity depends not only on the magnitude of the antibody response but also on its specificity and functionality. For vaccine development, strategies should aim to preferentially elicit Abs against quaternary E-dimer epitopes while limiting responses to enhancing epitopes like the fusion loop. In clinical practice, profiling epitope-specific Ab responses could contribute to identifying individuals at risk of severe disease and to the evaluation of vaccine or therapeutic efficacy. Future research should validate these signatures prospectively across other dengue virus serotypes and populations, and develop standardized assays for their application in surveillance, clinical management, and vaccine trials.

## Introduction

Half of the world population lives in areas at risk for dengue infection. In tropical and subtropical regions, humans co-exist with *Aedes* mosquitoes (mainly *Ae. aegypti* and *Ae. albopictus*) and the four genetically and antigenically distinct dengue virus serotypes (DENV1-4) co-circulate. Globally, DENV causes nearly 400 million new infections each year, of which 96 million manifests clinically, leading to ~22,000 deaths annually and the majority of affected individuals are children (Bhatt et al, 2013; Deng et al, 2024). Dengue cases have recently increased due to climate change and weather shifts, urbanization facilitating vector-human contact, and an increase in travel and population density (Abbasi, 2025; Bhatt et al, 2013). Infections with any of the four DENV serotypes are asymptomatic in up to 75% of cases, while ~25% of infected individuals develop clinical disease, ranging from classical dengue fever (DF) to severe forms such as dengue hemorrhagic fever (DHF) and dengue shock syndrome (DSS) (Katzelnick et al, 2017). There is an urgent need for the development of antiviral treatments, biomarkers of disease severity and new, highly effective vaccines.

Primary infection with any DENV serotype rarely causes severe disease but induces serotype-specific neutralizing antibodies (nAbs) that protect against homotypic reinfections, and cross-reactive nAbs which provide short-term (3 months to several years) protection against heterotypic DENV infections (Katzelnick et al, 2017; Salje et al, 2018). However, preexisting non-protective cross-reactive Abs can predispose individuals to symptomatic secondary infections with heterotypic DENVs and severe disease by facilitating viral entry into innate immune cells such as monocytes, macrophages and dendritic cells through Fcγ receptor (FcγR)-mediated uptake of IgG-DENV immune complexes (Bournazos et al, 2020; Halstead et al, 2010), a mechanism termed antibody-dependent enhancement (ADE). In addition, FcγR-dependent entry can suppress type I interferon signaling, dampen innate immune response, increase pro-inflammatory cytokine production, and impair early cellular and humoral immunity, thereby contributing to dengue immunopathogenesis (Chareonsirisuthigul et al, 2007; Hardy et al, 2025).

The humoral immune response is mainly directed to the DENV Envelope (E) protein. Each E protein monomer consists of three domains: domain I (EDI), domain II (EDII), and domain III (EDIII), which contribute to host cell attachment in the dimeric form and facilitate membrane fusion in the trimerized form (Lok et al, 2008). A conserved fusion loop (FL) within EDII plays a crucial role in membrane fusion, while EDIII is proposed to serve as the viral attachment site to host cells (Rey et al, 2018). In mature virus particles, the E protein is organized into 90 homodimers arranged in an antiparallel head-to-tail fashion. At the interface between two E-monomers, a highly conserved conformational epitope known as the E-dimer epitope (EDE) is formed (Dejnirattisai et al, 2016; Rouvinski et al, 2015). Recombinantly produced soluble E (sE) lacks the C-terminal transmembrane anchor and a segment immediately preceding it. Stabilized sE-dimers, where two mutations were introduced to construct two disulfide bonds containing sE in a dimeric form, have been used to omit exposure of the fusion loop and to characterize monoclonal antibodies targeting the sE-dimer interface (Rouvinski et al, 2015; Sharma et al, 2021).

Abs targeting EDIII are mainly serotype-specific neutralizing Abs with minimal or no ADE potential, but they constitute only a small fraction of the overall Ab response following natural infection (Gallichotte et al, 2015; Wahala et al, 2009). Depleting anti-EDIII Abs from human serum has been shown to reduce neutralization across all DENV serotypes by up to 3.5-fold (Medina-Carrasco et al, 2023), while another study has reported minimal impact of EDIII-reactive Ab removal on neutralization or ADE (Chen et al, 2016). Panels of monoclonal Abs (mAbs) have been generated to study in depth the anti-E humoral response. mAbs targeting the FL are predominantly cross-reactive and weakly neutralizing, likely due to limited epitope accessibility, and have been shown to mediate ADE in both in vitro and in vivo models (Balsitis et al, 2010; Priyamvada et al, 2017; Rey et al, 2018). mAbs directed against the EDE epitope mediate both serotype-specific and cross-neutralizing activity against all four DENV serotypes as well as Zika virus (ZIKV), as demonstrated both in vivo and in vitro studies (Lim et al, 2021; Sharma et al, 2021; Shukla et al, 2020; Swanstrom et al, 2016). While epitope-specific mAbs responses, such as those targeting EDE, FL, or EDIII, have been characterized in vitro and in in vivo models, their direct association with clinical outcomes following natural infection, particularly in pediatric populations, remains poorly defined.

ADE is thought to be mediated by non-neutralizing or sub-neutralizing titers of anti-dengue antibodies. ADE occurs when neutralizing antibodies wane or when cross-reactive low-affinity antibodies and/or high-affinity antibodies targeting cryptic epitopes are present (Dejnirattisai et al, 2010; Katzelnick et al, 2017), and depends on the time, genetic distance and the order of previous infection with DENV of different serotypes (Bos et al, 2024; Wang et al, 2024). In the acute and critical phase of a post-primary DENV infection, the polyclonal humoral immune response will consist of both preexisting and newly formed anti-DENV antibodies. However, the proportions of anti-E antibodies targeting different epitopes on E and their association with protection or disease severity remains unresolved.

This study aims to characterize DENV E protein epitope-specific Abs and their association with disease outcomes during post-primary infection in two different cohorts. Using a microsphere-based immunoassay (MIA) and a targeted antibody purification approach, we dissected the functional properties of E protein epitope-specific Abs. We demonstrate that the proportion of FL-like antibodies correlates with disease susceptibility, whereas the proportion of sE-dimer-specific Abs determines disease severity in hospitalized patients. These findings have important implications for the development of new vaccine strategies.

## Results

### Kinetics and epitope specificity of anti-DENV2 E Abs during post-primary DENV2 infection

**Figure 1:**
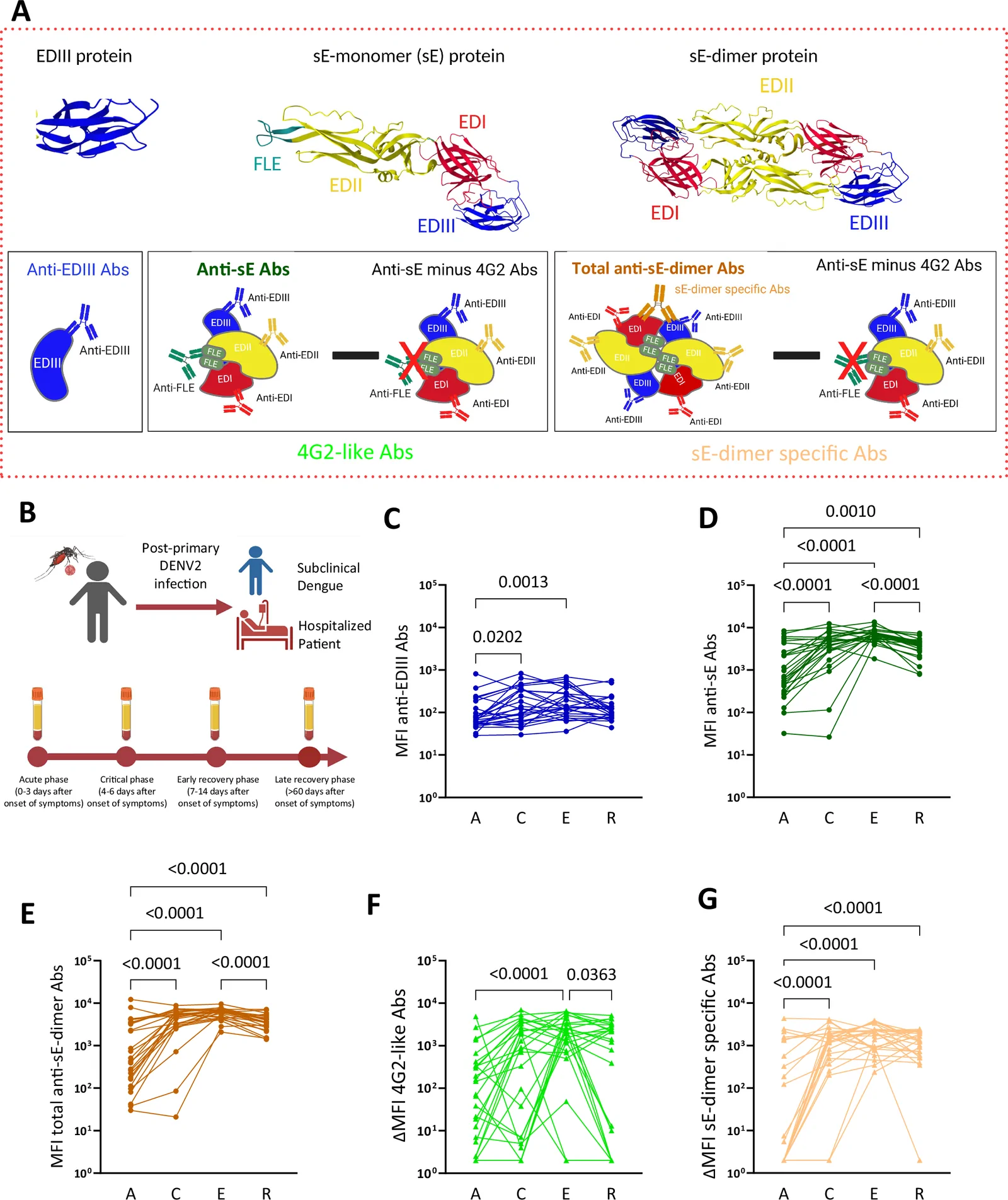
Evaluation of anti-DENV2 E antibody responses over time during post-primary DENV2 infection in the Cambodian cohort. (**A**) Schematic representation of DENV E protein (EDIII, sE, and sE-dimer) structure used to detect anti-DENV E antibody (Abs) subsets in DENV-infected individuals. mouse mAb clone 4G2 was used at a concentration of 10 μg/mL to block the FL epitope in sE. The total anti-sE-dimer Abs indicate all Abs binding to the sE-dimer, which include anti-EDI, EDII, EDIII Abs but not anti-FL Abs, as the FL is not exposed and partially mutated in the stabilized dimer (Lay et al, 2025). sE-dimer specific Abs indicate antibodies binding exclusively to the dimer, hence binding to quaternary epitopes. (**B**) Schematic representation of patient identification and sample collection, plasma samples were collected longitudinally from Cambodian children with confirmed post-primary DENV2 infection (DF: *n* = 22, DHF/DSS: *n* = 2, subclinical: *n* = 2), spanning the acute to recovery phases. Total IgG was purified from plasma of paired samples from across different infection phases (A = Acute; C = Critical; E = Early recovery; R = Recovery) to measure: (**C**) *anti-EDIII Abs* (*p* = 0.0202; acute phase Vs critical phase, and *p* = 0.0013; acute phase Vs early recovery phase) (**D**) *anti-sE Abs* (*p* < 0.0001 across different phases of infection), (**E**) *total anti-sE-dimer Abs* (*p* < 0.0001 across different phases of infection), (**F**) *Proportion of 4G2-like Abs in total anti-sE Abs* (*p* < 0.0001; acute phase Vs early recovery phase, and *p* = 0.0363; early recovery phase Vs recovery phase), and (**G**) *Proportion of sE-dimer specific Abs* in total anti-sE dimer Abs (*p* < 0.0001; acute phase Vs critical, early recovery, and recovery phases). Each line in the graphs represents DENV2 E epitope-specific Abs for an individual across the infection timeline. Data were shown as median fluorescence intensity (MFI) IgG or ∆MFI IgG (background-subtracted signal). Bonferroni’s multiple comparison test was used to compare responses across phases (**C**–**G**). Phases labeled on x axis: A acute phase (0–3 days after onset of symptoms), C critical phase (4–6 days after onset of symptoms), E early recovery phase (7–14 days after onset of symptoms), R recovery phase (2 months post onset of symptoms). All the experiments were run in a single. The schematic representations shown in (**A**, **B**) were created in Biorender. Source data are available online for this figure.

The presence of anti-DENV2 sE protein Abs was assessed using a MIA, a previously described sensitive and quantitative method to evaluate anti-E antibody subsets (Lay et al, 2025). Antibodies binding to recombinant, stabilized sE-dimer, sE monomer or EDIII domain were measured. Next, the proportion of epitope-specific antibodies was measured using binding comparison and blocking experiments; i.e. the proportion of anti-FL and sE-dimer specific antibodies within the total anti-sE antibodies were measured by blocking experiments using the pan-flavivirus anti-FL antibody clone 4G2 (Fig. 1A). Hence, total anti-sE-dimer Abs indicate all antibodies binding to the sE-dimer (including anti-EDI, EDII, EDIII antibodies but not anti-FL antibodies as the FL is not exposed and partially mutated in the stabilized dimer (Lay et al, 2025)), while sE-dimer specific Abs (not binding to EDIII) indicate antibodies binding exclusively to the dimer, hence binding to quaternary epitopes. 4G2-like Abs indicate antibodies binding to the fusion loop.

In order to evaluate the dynamic changes of each antibody subset, antibodies were measured in plasma collected from children who had confirmed post-primary DENV2 infection at the acute phase (0–3 days after onset of symptoms), critical phase (4–6 days after onset of symptoms), early convalescence (7–14 days after onset of symptoms) and late convalescence (2 months post onset of symptoms) (Fig. 1B). In the subclinical group who had no symptoms (*n* = 5), D0 and D3 samples were categorized as acute and critical phases, respectively. The amount of epitope-specific antibodies varied substantially between individuals in the acute phase and converged in the recovery phase. Amount of anti-sE, total anti-sE dimer, anti-FL and sE-dimer specific antibodies increased significantly over time and peaked during the critical and early convalescent phase (Fig. 1C–G).

**Figure 2:**
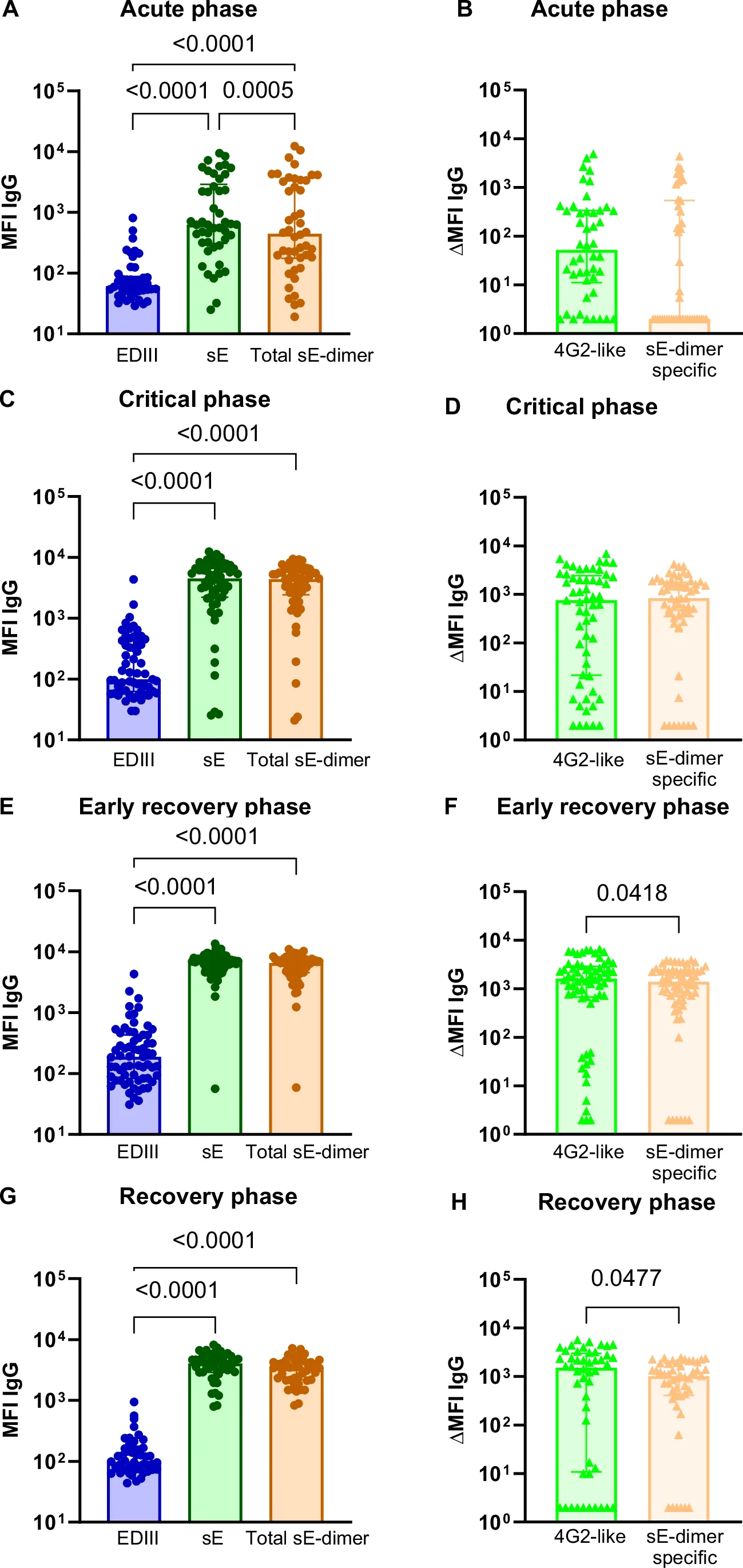
Measurement of epitope-specific antibodies across infection phases in the Cambodian cohort. Comparison of antibody (Ab) binding to various epitopes on the DENV2 recombinant E protein, including (**A**, **C**, **E**, **G**) EDIII (blue dots), sE (green dots), total sE-dimer (orange dots), and (**B**, **D**, **F**, **H**) proportion of 4G2-like Abs in total anti-sE Abs (light green triangles), and proportion of sE-dimer specific Abs in total sE-dimer Abs (light orange triangles) across sequential phases of dengue infection: (**A**,** B**) acute phase (*n* = 46, 0–3 days after onset of symptoms); p < 0.0001 comparison of anti-EDIII with anti-sE and total sE-dimer Abs and *p* = 0.0005 between anti-sE and total sE-dimer Abs (**C**,** D**) Critical phase (*n* = 57, 4–6 days after onset of symptoms); *p* < 0.0001 comparison of anti-EDIII with anti-sE and total sE-dimer Abs, (**E**,** F**) Early recovery phase (*n* = 65, 7–14 days after onset of symptoms); *p* < 0.0001 comparison of anti-EDIII with anti-sE and total sE-dimer Abs and *p* = 0.0418 between 4G2-like Abs and sE-dimer specific Abs, (**G**,** H**) Recovery phase (*n* = 48, 2 months post onset of symptoms); *p* < 0.0001 comparison of anti-EDIII with anti-sE and total sE-dimer Abs and *p* = 0.0477 between 4G2-like Abs and sE-dimer specific Abs. Data were shown as median fluorescence intensity (MFI) IgG or ∆MFI IgG. Each dot or triangle represents an individual. Box-and-whisker plots show the median and interquartile range. All the experiments were run in a single. The Wilcoxon test was used for pairwise comparison (**B**, **D**, **F**, **H**), and the Bonferroni multiple comparison test was used for multiple comparisons (**A**, **C**, **E**, **G**). Source data are available online for this figure.

**Figure EV1:**
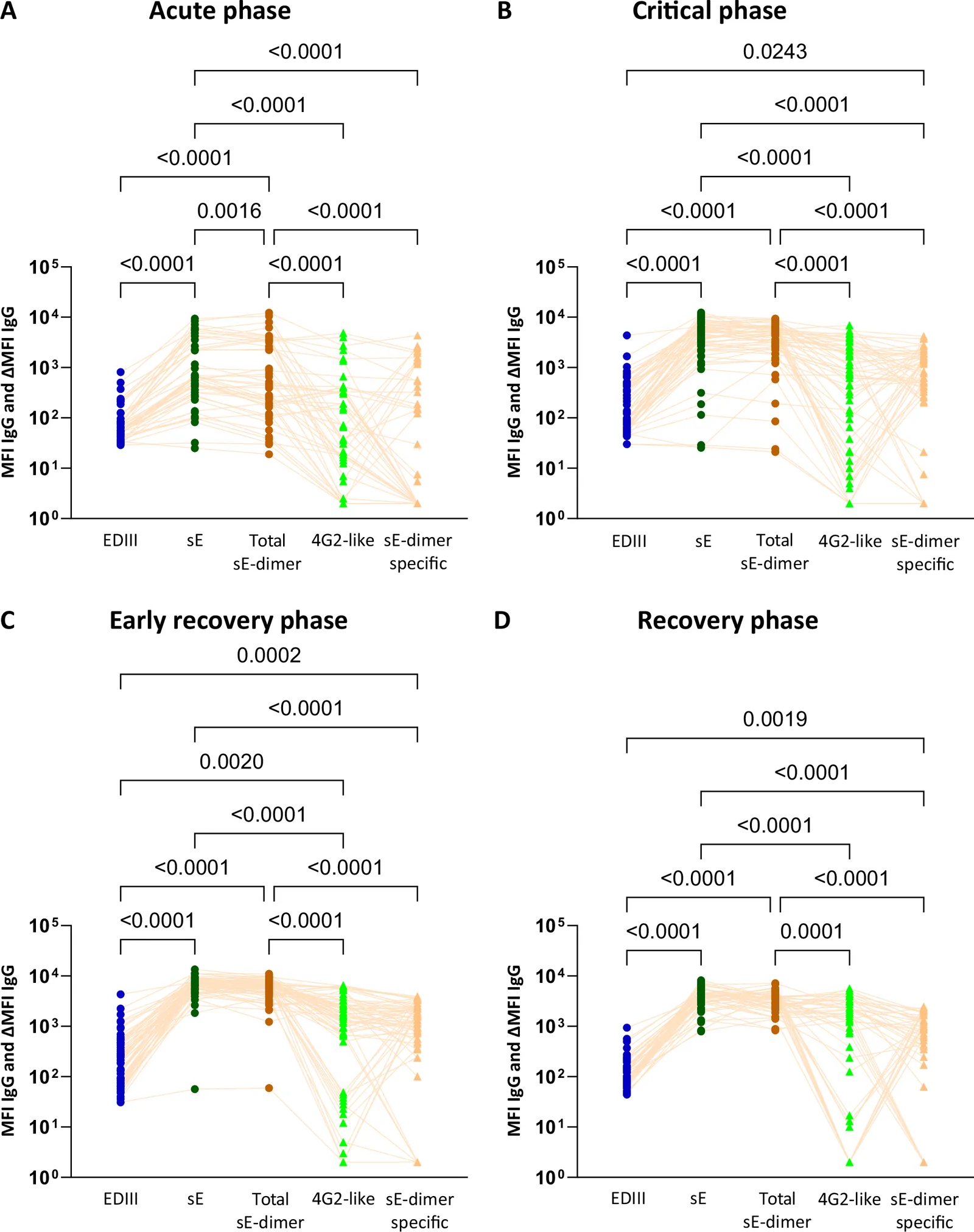
Distribution of anti-E antibodies targeting diverse epitopes across different infection phases in the Cambodian cohort. DENV E protein epitope-specific Abs in the plasma of dengue patients who had confirmed secondary infection with DENV2 were detected using MIA. Plasma samples were collected during (**A**) the acute phase (0–3 days after onset of fever; *n* = 46) (all *p* < 0.0001 except anti-sE Vs total sE-dimer Abs (*p* = 0.0016)), (**B**) critical phase (4–6 days after onset of fever; *n* = 57) (all *p* < 0.0001 except anti-EDIII Vs sE-dimer specific Abs (*p* = 0.0243)), (**C**) early recovery phase (7–14 days after onset of fever; *n* = 65) (all *p* < 0.0001 except anti-EDIII Vs sE-dimer specific Abs (*p* = 0.0002) and 4G2-like Abs (*p* = 0.0020)), and (**D**) the recovery phase (60 days after onset of fever; *n* = 48) (all *p* < 0.0001 except anti-EDIII Vs sE-dimer specific Abs (*p* = 0.0019) and Total sE-dimer Vs 4G2-like Abs (*p* = 0.0001)). Comparison of the Abs binding to EDIII (Blue dots), sE (green dots), total sE-dimer (orange dots), proportion of 4G2-like Abs (light green triangles), and proportion of sE-dimer specific Abs (light orange triangles). Each line represents an individual, and the data shown as MFI IgG or proportion MFI IgG (∆MFI IgG). Paired Bonferroni’s multiple comparison test was used to compare multiple groups in panels (**A**–**D**). All experiments were performed in single.

Next, we investigated the distribution of Abs targeting distinct epitopes on the DENV2 E protein at different phases of infection (Figs. 2 and EV1). Anti-EDIII Ab concentrations were significantly lower than those of anti-sE and total anti-sE-dimer Abs at all timepoints measured (*p* < 0.0001) (Fig. 2A–H). During the early recovery and recovery phase, anti-FL Abs were increased compared to sE-dimer-specific Abs (Fig. 2F,H). Notably, the levels of anti-DENV sE Ab subset displayed substantial inter-individual variability, particularly during the acute and critical phases (Fig. 2A,C), suggesting heterogeneity in immune recognition or differences in epitope exposure and response kinetics among individuals.

### The association of anti-E protein epitope-specific Abs with disease outcome

**Figure 3:**
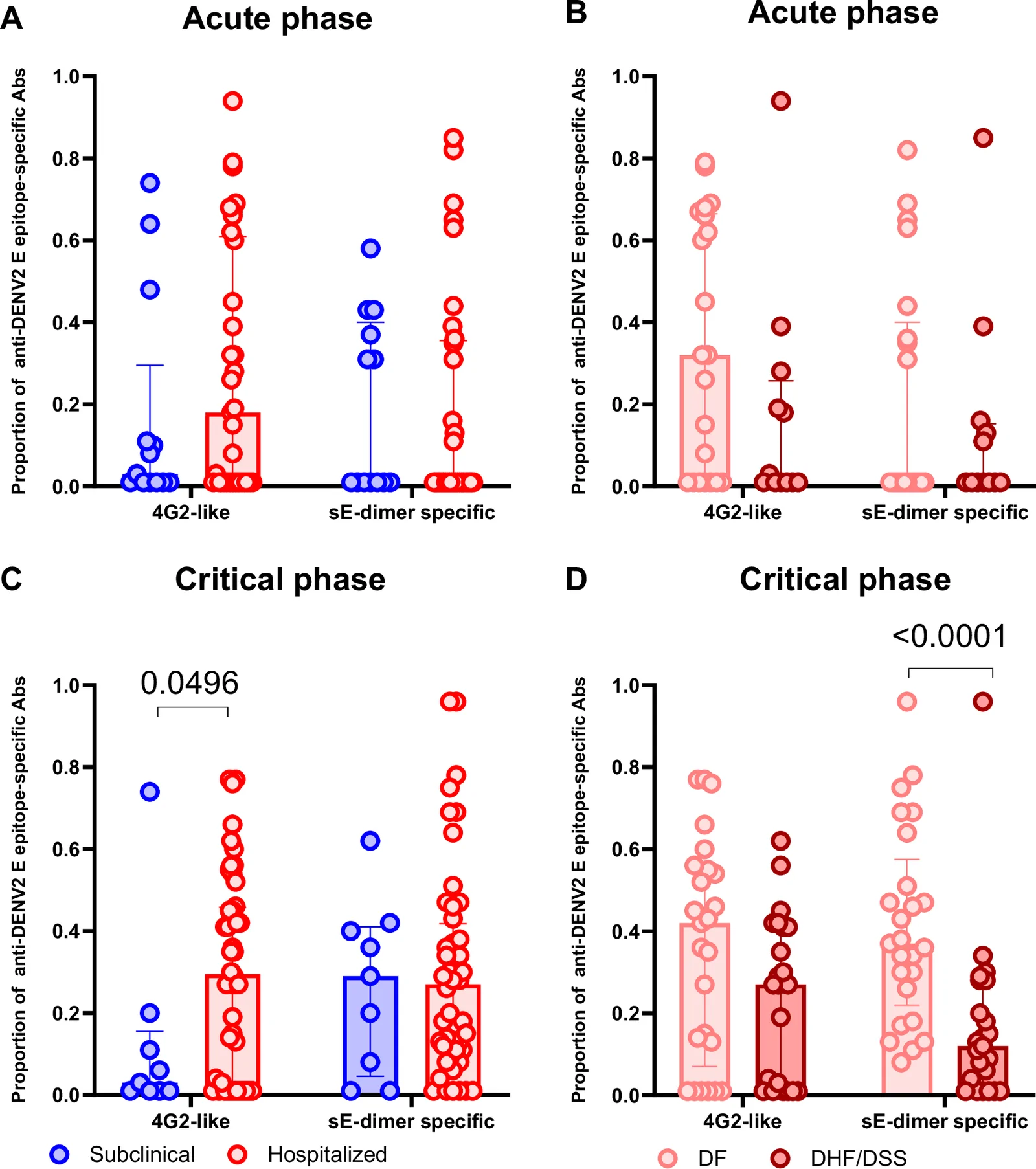
Comparison of the proportions of DENV2 4G2-like and sE-dimer specific antibodies across different disease outcomes during the acute and the critical phase of disease in the Cambodian cohort. Comparison of the proportion of 4G2-like Abs in total anti-sE Abs, and proportion of sE-dimer specific Abs in total sE-dimer Abs were made between subclinical cases (blue, *n* = 13) and hospitalized patients (red, *n* = 33), and dengue fever (DF) (light red, *n* = 21), and dengue hemorrhagic fever (DHF)/dengue shock syndrome (DSS) cases (dark red, *n* = 12) during the (**A**,** B**) acute phase and subclinical cases (blue, *n* = 9) and hospitalized patients (red, *n* = 48); 4G2-like Abs, *p* = 0.0496, and dengue fever (DF) (light red, *n* = 25), and dengue hemorrhagic fever (DHF)/dengue shock syndrome (DSS) cases (dark red, *n* = 23); sE-dimer specific Abs, *p* < 0.0001 during the (**C**,** D**) critical phase. Each dot represents an individual; box-and-whisker plots indicate median and interquartile range. The Mann–Whitney test was used to compare two groups. All experiments were performed in single. Source data are available online for this figure.

**Figure EV2:**
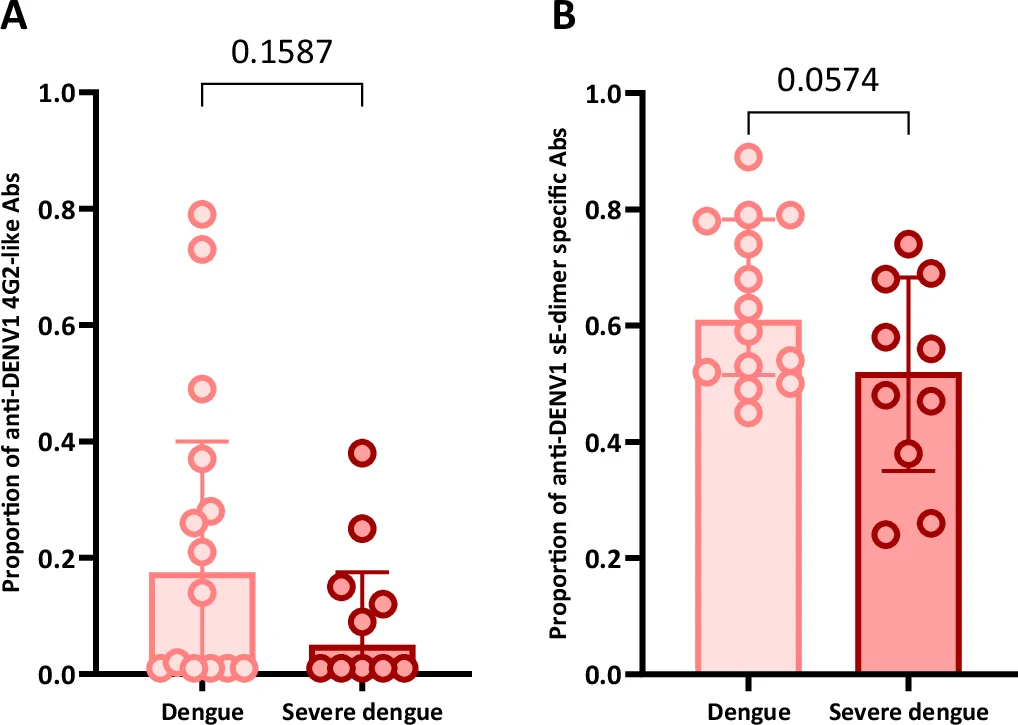
Comparison of the proportions of 4G2-like and sE-dimer specific antibodies in Colombian hospitalized patients. Plasma samples were collected from Colombian patients with confirmed secondary DENV1 infection at 2–7 days after the onset of symptoms. Anti-DENV1 antibody (Ab) subsets were measured using microsphere-based immunoassay (MIA) using DENV1-derived sE, sE-dimer and EDIII recombinant proteins. (**A**) Proportion of 4G2-like Abs (*p* = 0.1587), and (**B**) proportion of sE-dimer specific Abs (*p* = 0.0574) were compared between dengue (*n* = 14) and severe dengue (*n* = 10) cases. Each dot represents an individual; box-and-whisker plots display the median and interquartile range. Statistical comparison between groups was performed using the unpaired *T*-test.

Anti-DENV sE Abs consist of a polyclonal pool of antibodies mediating neutralization, ADE and other IgG effector functions, depending on the epitope targeted, concentration and IgG Fc characteristics. In order to understand the contribution of E protein epitope-specific antibodies to disease outcome, we analysed the proportion of anti-FL antibodies and sE-dimer-specific antibodies in patients with different clinical outcomes after infection during the acute and critical phases of disease, when the virus is still circulating. In the acute phase, a similar percentage of both subclinical and hospitalized patients had detectable levels of sE-dimer specific antibodies (6/13 (46%) in the subclinical group versus 13/33 (39%) in the hospitalized group). (Fig. 3A,B). No differences in antibody proportions were observed between both groups at this timepoint. Subsequently, we stratified the hospitalized patients by disease severity following the WHO 1997 classification criteria, but no differences were observed between DF and DHF/DSS patients. In the critical phase however, the proportion of anti-FL Abs was significantly higher in hospitalized patients compared to those with subclinical infection (*p* = 0.0496, 0.03 (0.01–0.15) versus 0.29 (0.01–0.45)), while there was no difference in the proportion of sE-dimer specific Abs (Fig. 3C). Considering hospitalized patients only, the proportion of sE-dimer-specific Abs was significantly lower in DHF/DSS cases compared to DF patients (*p* < 0.0001, 0.37 (0.22–0.57) versus 0.12 (0.01–0.28)) (Fig. 3D). To validate these findings, we analysed the epitope-specific Ab profiles in an independent cohort undergoing a RT-PCR confirmed post-primary DENV1 infection from Colombia using DENV1-derived recombinant E proteins (Ghita et al, 2023). Indeed, also in this cohort, the proportion of DENV1 sE-dimer specific Abs was higher in DF patients than in those with DHF/DSS patients, albeit the comparison did not reach significance (*p* = 0.0574, 0.61 (0.51–0.78) versus 0.52 (0.35–0.68)) (Fig. EV2). Taken together, our findings suggest that a higher proportion of anti-FL Abs in the critical phase is associated with symptomatic infection, while a higher proportion of sE-dimer-specific Abs is protective against severe dengue disease. These data provide evidence of both protective and pathogenic roles for anti-DENV E Abs in human dengue.

### Purity and functional characteristics of isolated DENV2 anti-E protein epitope-specific Abs

**Figure EV3:**
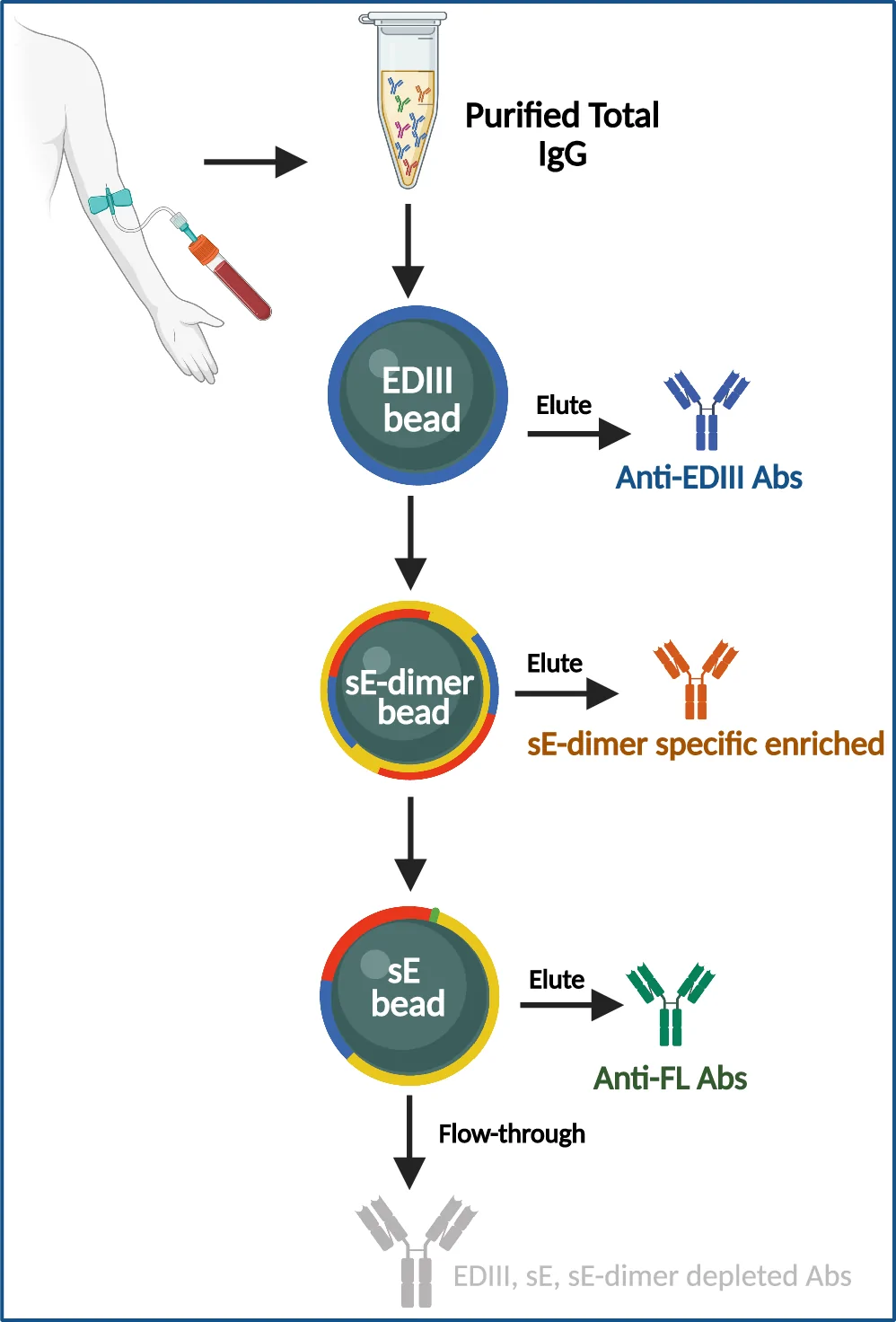
Scheme of DENV E protein Epitope-specific Abs isolation from human plasma. Total IgG was isolated from the plasma of DENV2-infected individuals and used for epitope-specific antibody purification. Site-specific biotinylated DENV2 E protein antigens (EDIII, sE, and sE-dimer) were immobilized on streptavidin-coated resin beads. The negative and positive selection were used, and purification of the isolated fraction were confirmed by MIA. Details of the protocol and validation of the isolation has been published before (Lay et al, 2025). All experiments were performed in single.

**Figure 4:**
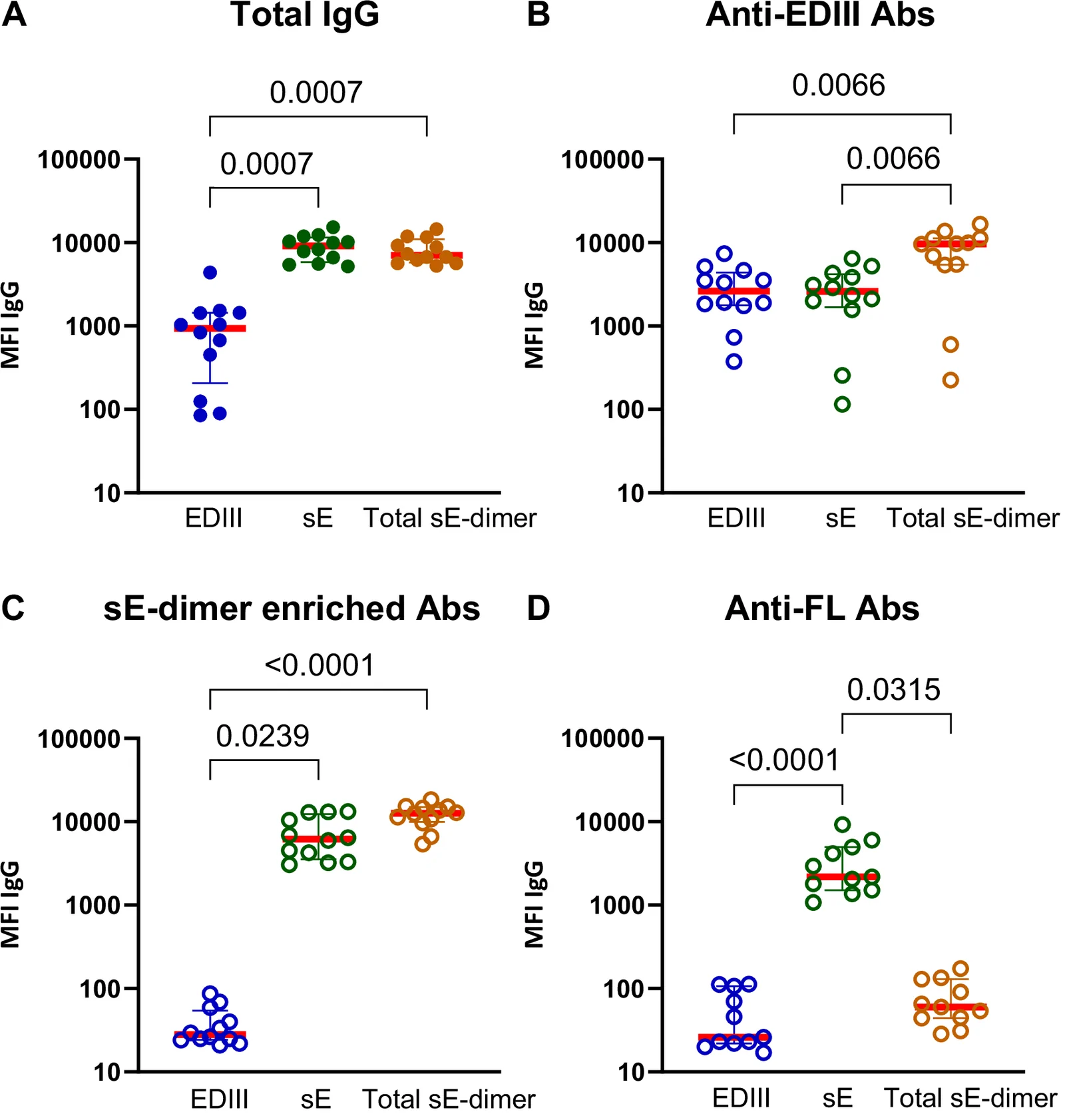
Purification of DENV2 E protein epitope-specific antibodies from human plasma. Total IgG isolated from 12 DENV2-seropositive individuals during the critical phase (4–6 days after onset of symptoms) was subjected to epitope-specific purification (as previously described in Lay et al, 2025. The binding properties of antibodies (Abs) before and after isolation were assessed by microsphere-based immunoassay (MIA) using recombinant DENV E proteins. Shown are binding profiles for (**A**) total IgG (*n* = 12); *p* = 0.0007 (anti-EDIII Abs Vs sE and total sE-dimer Abs), (**B**) anti-EDIII Abs (*n* = 12); *p* = 0.0066 (total sE-dimer Vs EDIII and sE Abs), (**C**) sE-dimer specific enriched Abs (*n* = 12); *p* = 0.0239 (anti-EDIII Vs sE Abs) and *p* < 0.0001 (anti-EDIII Vs total sE-dimer Abs), and (**D**) anti-FL Abs (*n* = 11); *p* < 0.0001 (anti-EDIII Vs sE Abs) and *p* = 0.0315 (anti-sE Vs total sE-dimer Abs). Each dot represents the Ab binding signal as median fluorescence intensity (MFI) IgG. Whiskers show median and interquartile range. The Friedman test was used to compare multiple groups, and experiments were performed in single. Source data are available online for this figure.

To analyse the functionality of the anti-E protein epitope-specific Abs, we isolated anti-EDIII, anti-FL, and sE-dimer specific Ab fractions from polyclonal IgG using a previously established and validated protocol (Lay et al, 2025), (Fig. EV3). Twelve human plasma samples obtained from patients undergoing post-primary DENV2 infection during the critical phase were used for epitope-specific Ab purification, as this is the phase showing a high amount of anti-E antibodies (Fig. 1B–D). As shown in Fig. 4A, purified total IgG of all 12 patients showed reactivity to all tested DENV2 E proteins, with higher amounts of anti-sE and anti-sE dimer antibodies compared to anti-EDIII antibodies (*p* = 0.0007; 936 MFI (206.3–1442) versus 9142 MFI (5831–11510) and *p* = 0.0077; 936 MFI (206.3–1442) versus 7040 (5784–11022)). After purification, the fraction containing anti-EDIII Abs bound to all three recombinant antigens (Fig. 4B). As expected, the sE-dimer specific Abs preferentially bound to sE-dimer over sE and did not bind to EDIII (*p* = 0.0239; 28 MFI (24.25–54.25) versus 6190 MFI (3539–12326) and *p* < 0.0001; 28 MFI (24.25–54.25) versus 12637 MFI (9953–14964)) (Fig. 4C). In contrast, anti-FL Abs showed significantly higher binding to sE compared to the other proteins, the only recombinant antigen where the FL is exposed (*p* < 0.0001; 2188 MFI (1505–4962) versus 26 MFI (22–107) compared to EDIII and *p* = 0.0315; 2188 MFI (1505–4962) versus 66 MFI (44–129.5) compared to sE-dimer) (Fig. 4D). These results confirm the successful purification of anti-EDIII and anti-FL Abs, as well as the enrichment of sE-dimer specific Abs from human plasma.

**Figure 5:**
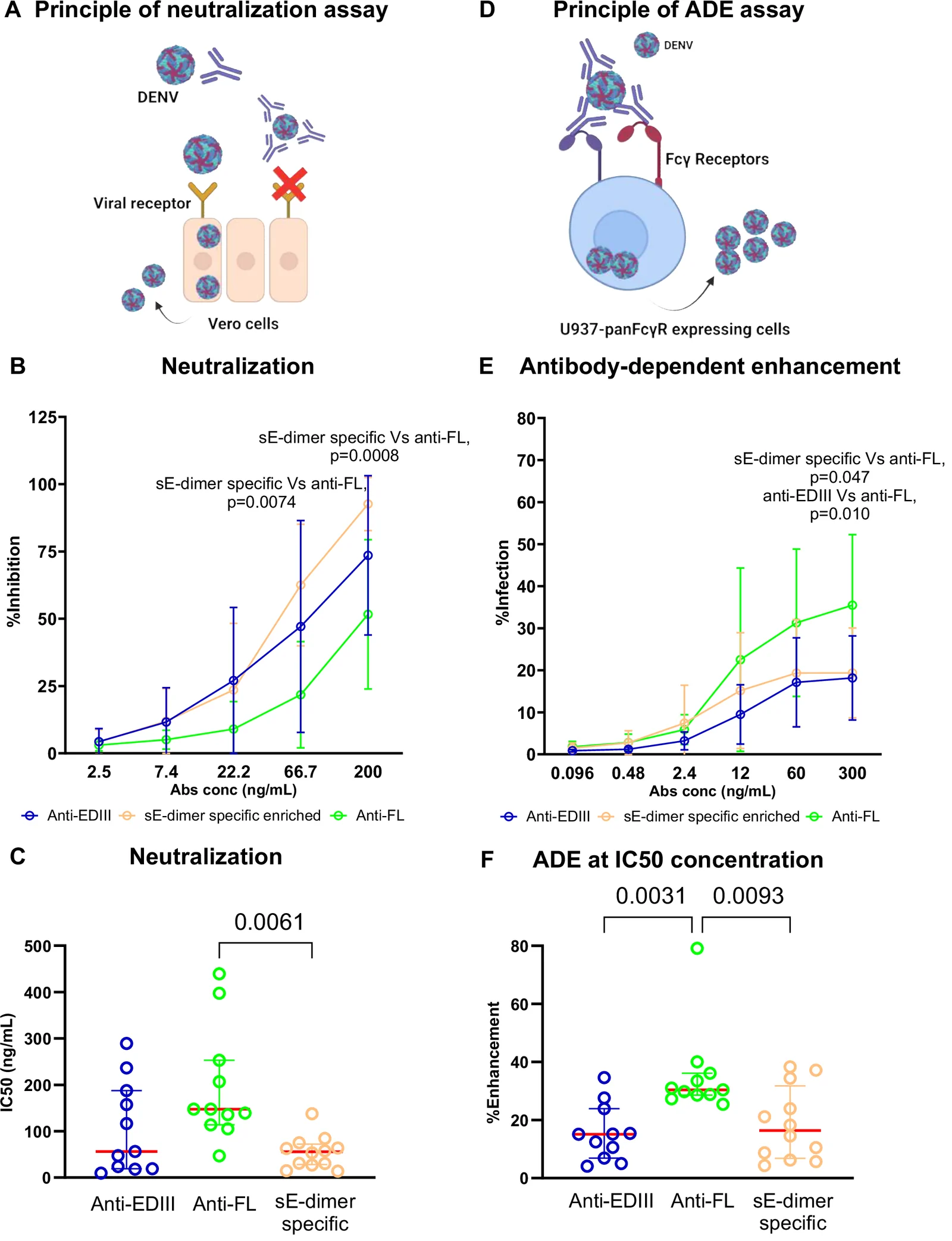
Functional characterization of anti-DENV2 E protein epitope-specific antibody fractions. Anti-EDIII (blue line, *n* = 11), anti-FL (light green line, *n* = 11) and sE-dimer-specific enriched (light orange line, *n* = 12) antibody (Ab) fractions were isolated from plasma of DENV2-infected individuals during the critical phase (4–6 days after onset of symptoms). Their functional properties against DENV2 were evaluated using in vitro assays. Due to the low amount of epitope-specific antibodies, experiments were performed in single. In one individual, insufficient amounts of anti-EDIII antibodies could be purified, while in another individual, insufficient amounts of anti-FL antibodies could be purified. (**A**) Schematic representation of the neutralization assay principle. Created in Biorender. (**B**) Comparison of neutralizing activity of the isolated Ab fractions across a range of concentrations. Error bars indicate mean and standard deviation (**C**) Comparison of the concentration required to achieve 50% infection inhibition for each Ab fraction (*p* = 0.0061; comparison between anti-FL Abs and sE-dimer specific Abs). Each dot indicates one donor, the whisker median and interquartile range. (**D**) Schematic representation of the antibody-dependent enhancement (ADE) assay. Created in Biorender. (**E**) Comparison of enhancement activity across concentrations for each Ab fraction (anti-FL Abs Vs anti-EDIII Abs (*p* = 0.0031) and sE-dimer specific Abs (*p* = 0.0093)). Error bars indicate mean and standard deviation. The percentage of inhibition was calculated following the formula: % inhibition = 100 – ((% infection in the presence of Abs ×  100)/% infection in the absence of Abs). (**F**) Comparison of enhancement activity in different antibody fractions at the concentration of IC50. The percentages of enhancement and IC50 concentration were used to calculate the enhancement of infectivity at IC50 concentration using a sigmoidal, 4PL concentration base model. Nonparametric Dunn’s test was used for multiple comparisons across different concentrations (**B**,** E**), while the Bonferroni test was used for group-level multiple comparisons (**C**,** F**). Source data are available online for this figure.

**Figure EV4:**
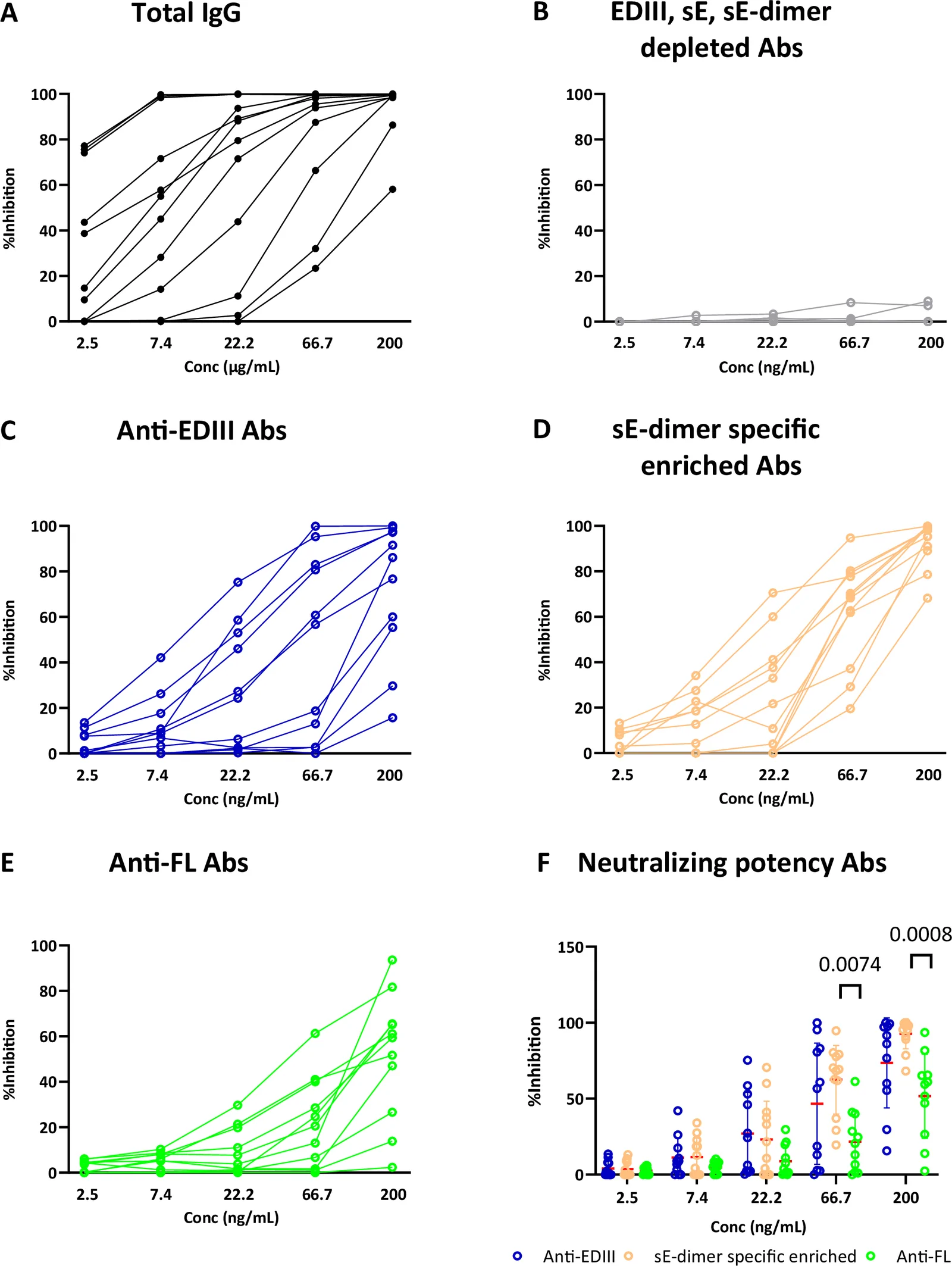
Neutralizing capacity of isolated anti-DENV E protein antibody fractions. Neutralizing activity was assessed using a flow cytometry-based assay in which Vero CCL-81 cells were infected with DENV2 in the presence or absence of serially diluted antibody (Ab) fractions. Due to the low amount of epitope-specific antibodies, experiments were performed in single. In one individual, insufficient amounts of anti-EDIII antibodies could be purified, while in another individual, insufficient amounts of anti-FL antibodies could be purified. Shown are the neutralization profiles for: (**A**) total IgG (*n* = 12), (**B**) EDIII, sE, and sE-dimer depleted Abs (*n* = 12), (**C**) anti-EDIII Abs (*n* = 11), (**D**) sE-dimer specific enriched (*n* = 12), and (**E**) anti-FL Abs (*n* = 11). (**F**) Comparison of neutralizing potency across isolated Ab fractions anti-EDIII (blue), sE-dimer-specific enriched (light orange) and anti-FL Abs (light green) at various concentrations. Percentage of inhibition was calculated following formula % inhibition =  100 – ((% infection in the presence of Abs × 100)/% infection in the absence of Abs). Each dot represents an individual, and whisker plots show the mean and standard deviation. The Dunn’s multiple test was used for multiple comparisons across Ab fractions at each concentration. All experiments were performed in single.

**Figure EV5:**
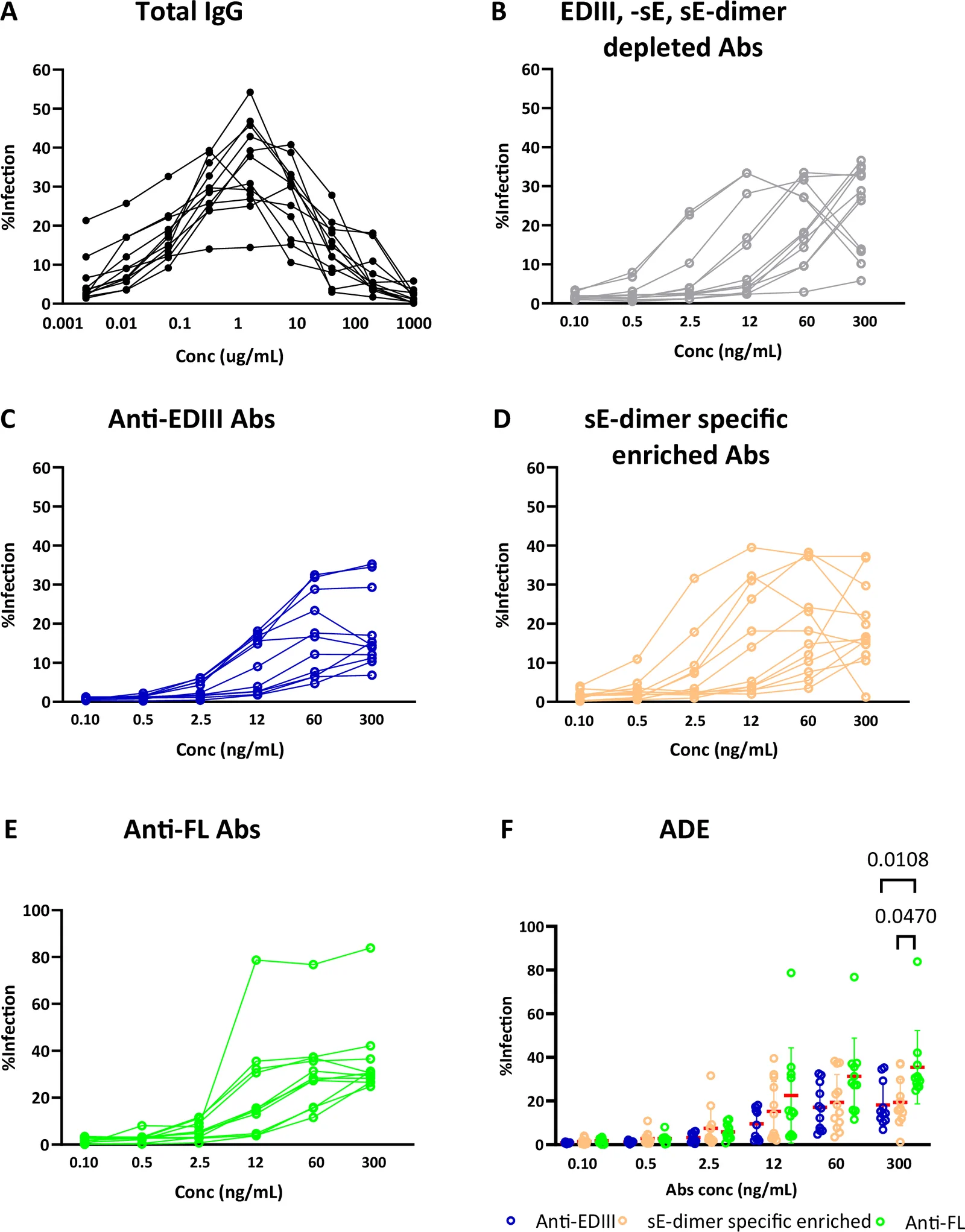
Antibody-dependent enhancement (ADE) activity of isolated anti-DENV E protein antibody fractions. The enhancing potential of isolated antibody (Ab) fractions was assessed using an in vitro ADE assay with U937-panFcγR expressing cells. Due to the low amount of epitope-specific antibodies, experiments were performed in single. In one individual, insufficient amounts of anti-EDIII antibodies could be purified, while in another individual, insufficient amounts of anti-FL antibodies could be purified. The cells were infected with DENV2 in the absence or presence of serially diluted Ab fractions: (**A**) total IgG (*n* = 12), (**B**) EDIII, sE, sE-dimer depleted Abs (*n* = 12), (**C**) anti-EDIII Abs (*n* = 11), (**D**) sE-dimer specific enriched Abs (*n* = 12), and (**E**) anti-FL Abs (*n* = 11). (**F**) Comparison of ADE activity among anti-EDIII (blue; *n* = 11), sE-dimer-specific enriched (light orange; *n* = 12), and anti-FL (light green; *n* = 11) Ab fractions across a range of various concentrations. Each line represents the percentage of infected U937 cells induced by Ab fractions from an individual donor. The whisker plots show the mean and standard deviation. The Nonparametric Dunn’s test was used for multiple comparisons between the Ab fraction at each concentration.

**Figure 6:**
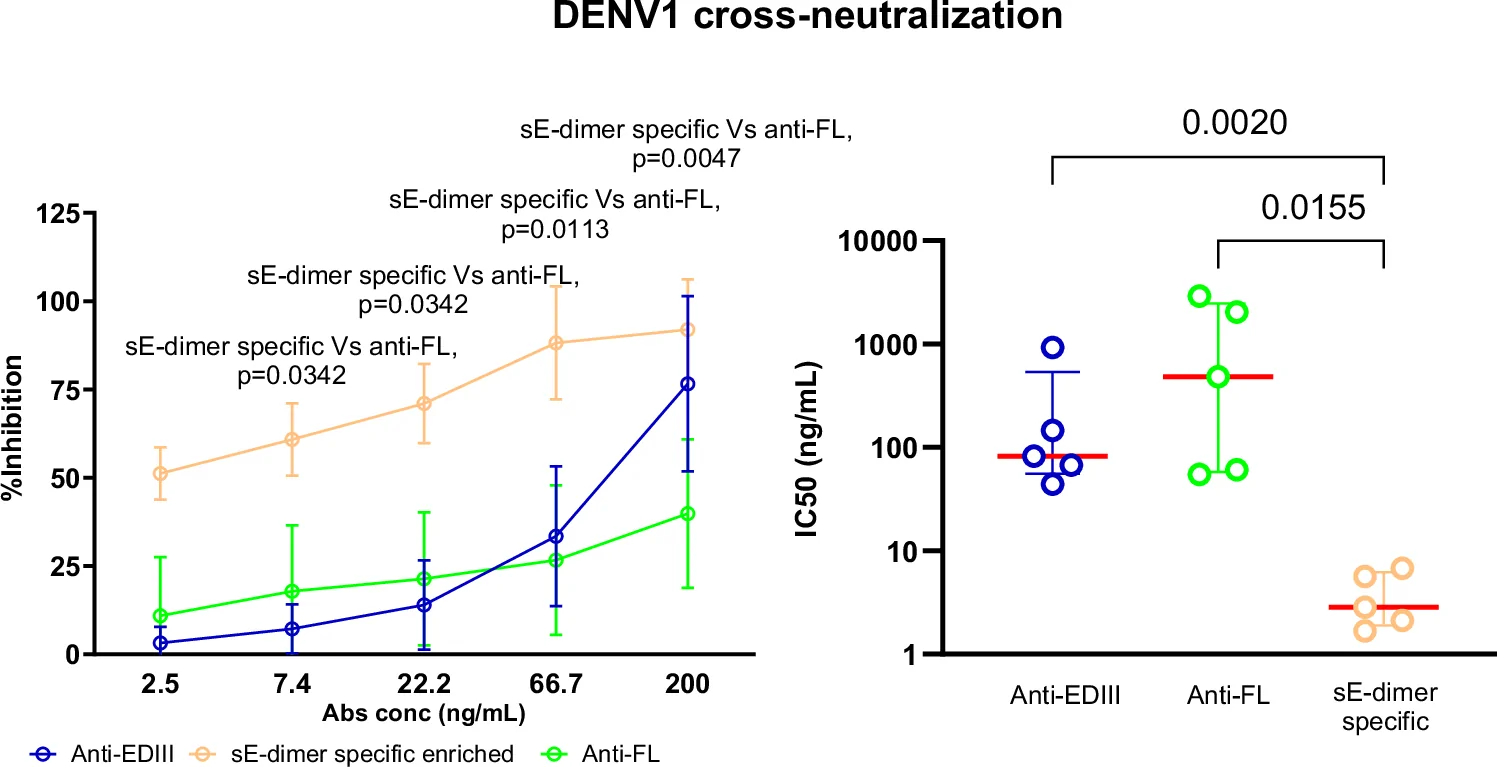
DENV1 cross-neutralization potency of anti-DENV2 E protein epitope-specific antibody fractions. Anti-EDIII (blue line, *n* = 5), anti-FL (light green line, *n* = 5), and sE-dimer-specific enriched (light orange line, *n* = 5) antibody (Ab) fractions were isolated from plasma of DENV2-infected individuals during the critical phase (4–6 days after onset of symptoms). Their functional properties against DENV2 were evaluated using in vitro assays. Due to the low amount of epitope-specific antibodies, experiments were performed in single. Comparison of neutralizing activity of the isolated Ab fractions across a range of concentrations. Error bars indicate mean and standard deviation. Comparison of the concentration required to achieve 50% infection inhibition for each Ab fraction (sE-dimer specific Abs vs anti-FL Abs (*p* = 0.0155) and anti-EDIII Abs (*p* = 0.0020). Each dot indicates one donor, the whisker median and interquartile range. Percentage of inhibition was calculated following formula: % inhibition = 100 – ((% infection in the presence of Abs × 100)/% infection in the absence of Abs). Nonparametric Dunn’s test was used for multiple comparisons across different concentrations, while the Bonferroni test was used for group-level multiple comparisons. Source data are available online for this figure.

We next evaluated the neutralizing and ADE capacity of the isolated Ab fractions against DENV2 using a flow cytometry-based assay (Fig. 5). As expected, total IgG and isolated Ab fractions exhibited both dose-dependent neutralizing and enhancing activities (Figs. EV4 and 5). However, depletion of EDIII, sE and sE-dimer Abs completely abolished the neutralization capacity but retained ADE at certain concentrations (Figs. EV4B and EV5B). sE-dimer specific enriched Abs exhibited significantly higher neutralization compared to anti-FL Abs purified from human plasma at concentrations ranging from 66.7 to 200 ng/ml (Fig. 5B). Moreover, anti-FL Abs required significant higher Ab concentrations to achieve 50% inhibition compared to the sE-dimer specific enriched fraction (*p* = 0.0061; 147.4 ng/ml (113.5–253.1) versus 55.6 ng/ml (28.0–72.1)) (Fig. 5C). Conversely, FL-targeting Abs induced more infection than anti-EDIII and sE-dimer specific enriched Abs fraction in the U937-panFcγR expressing cells at concentrations of 300 ng/ml (*p* = 0.0470; 30.5% (26.6–36.5) versus 16.3% (12.6–27.8), *p* = 0.0108; 30.5% (26.6–36.5) versus 14.4% (11.2–29.3) (Fig. 5E). In order to understand if different fractions exhibited different ADE potential at the same neutralization titers, we evaluated the ADE potential at the concentration where 50% of the virus is neutralized (IC50) for each fraction. Interestingly, at the IC50 concentration the anti-FL Abs exhibited higher infection enhancement compared to anti-EDIII and sE-dimer-specific enriched Abs fraction (*p* = 0.0031; 30.4% (28.6–36.1) versus 15.1% (6.9–23.9), and *p* = 0.0093; 30.4% (28.6–36.1) versus 16.4% (6.8–31.7) (Fig. 5F). As antibodies targeting quaternary epitopes have been reported to be potentially cross-neutralizing, we investigated the neutralization potential of the purified fractions against DENV1 (Dejnirattisai et al, 2015; Sharma et al, 2021). Due to the low amount of purified antibody fractions available, we were only able to test purified fractions of five individuals. Interestingly, sE-dimer-specific enriched Abs exhibited significantly higher cross-neutralizing potency against DENV1 compared to other epitope-specific Ab fractions (anti-EDIII vs sE-dimer specific: *p* = 0.0020, 82.29 ng/ml (55.61–533.6) versus 2.83 ng/ml (1.89–6.21), anti-FL versus sE-dimer specific: *p* = 0.0155, 481.1 ng/ml (57.87–2469) versus 2.83 ng/ml (1.89–6.21) (Fig. 6)

Our data highlight the functional heterogeneity of DENV2 E epitope-specific Abs and show that Abs targeting the sE-dimer epitope may provide a favorable balance between potent neutralization and reduced enhancement potential, while antibodies targeting the FL induce increased infection of FcγR expressing cells.

## Discussion

The global rise in dengue cases, driven by climate change, urbanization, and the geographic expansion of *Aedes* mosquitoes (Abbasi, 2025), underscores the urgent need to deepen our understanding of the immunological determinants that mediate protection versus pathogenesis, especially in pediatric populations. Children represent a major proportion of severe dengue cases (Deng et al, 2024; Verhagen and de Groot, 2014), and their immune responses may differ from those of adults. While previous studies have described the role and function of preexisting anti-DENV Abs in shaping the outcome of subsequent infections (Katzelnick et al, 2017; Salje et al, 2018; Balingit et al, 2024; Dias et al, 2025; Laoprasopwattana et al, 2005; Ngono and Shresta, 2018), limited data exist on the kinetics, specificity, and functional implications of Abs targeting distinct epitopes on the DENV E protein, particularly in the course of a post-primary infection, and their association with clinical severity.

Hence, we investigated the potential roles of epitope-specific antibody responses directed against distinct regions of the DENV2 E protein in a Cambodian pediatric population, focusing on their contribution to protection or disease progression. We demonstrated that anti-DENV2 E Abs increase over time following natural post-primary DENV2 infection and declined two months post-infection, consistent with previous studies (Muller et al, 2025; Vo et al, 2022). This pattern reflects expected adaptive immune kinetics, driven by antigen-induced B cell activation, immunoglobulin class switching, and affinity maturation (Wong et al, 2020). In addition, during heterotypic secondary DENV infection, cross-reactive memory B cells (MBCs) induced by the previous infection are preferentially reactivated and can dominate over responses specific to the current serotype (Priyamvada et al, 2016). Consequently, the overall affinity of anti-DENV Abs gradually shifts toward the infecting serotype as immune maturation progresses (Puschnik et al, 2013). By evaluating Ab responses to various E protein epitopes upon post-primary DENV2 infection, we found that of the amount of Abs targeting EDIII consistently remain lower than those targeting sE and the conformational sE-dimer across all infection phases. This observation aligns with previous findings indicating that anti-EDIII Abs constitute only a small fraction of the overall response and contribute minimally to the overall serum neutralization, possibly due to limited epitope accessibility or unfavorable conformational presentation during natural infection(Chen et al, 2016; Crill et al, 2009; Dejnirattisai et al, 2010; Wahala et al, 2009).

Notably, we observed substantial inter-individual variability, particularly during the acute and critical phases. In the critical phase, hospitalized patients had higher levels of anti-FL Abs than individuals with subclinical DENV2 infection. Such Abs typically recognize the FL epitope, which is conserved amongst all 4 dengue serotypes and other flaviviruses, and are known to be cross-reactive but weakly neutralizing (Beltramello et al, 2010; Dejnirattisai et al, 2010; Santos-Peral et al, 2024). Their increase in patients hospitalized for dengue may indicate their role in determining disease susceptibility, possibly through ADE. In contrast, sE-dimer-specific Abs, which target quaternary epitopes and are more likely to be broadly neutralizing, were more abundant in DF patients than in those with DHF/DSS, indicating that these antibodies could contribute to protection from severe disease. We observed similar directional trend across adult and children’s cohorts with different infecting serotypes and genetic background of patients, yet these association did not reach statistical significance in a cohort of adult Columbian patients infected with DENV1, probably due to the small sample size. Our findings are consistent with a recently published study showing that EDE-like Abs are associated with reduced dengue disease risk and mediate protective effects through neutralization and favorable binding profiles in a longitudinal patient cohort (Mpingabo et al, 2025).

To elucidate the mechanistic basis of these associations, we isolated anti-EDIII Abs, anti-FL Abs, and enriched sE-dimer-specific Abs fractions from polyclonal IgG and evaluated their functional properties using cell-based in vitro assays. The fractions enriched for sE-dimer specific Abs demonstrated significantly increased neutralization potency against both DENV1 and DENV2 than those purified Abs targeting the FL epitope. Conversely, FL-targeting Abs exhibited a markedly higher capacity to mediate infection of FcγR expressing cells. This is in agreement with earlier reports showing that recombinant mAbs targeting quaternary epitopes on DENV E protein confer strong cross-neutralization but limited ADE, whereas FL-specific mAbs tend to promote infection enhancement with limited neutralizing ability (Barba-Spaeth et al, 2016; Dejnirattisai et al, 2016; Rouvinski et al, 2017; Smith et al, 2012; Thomas et al, 2020; Wahala et al, 2009).

We did not observe any differences in neutralization potential between anti-EDIII Abs and the fraction enriched for sE-dimer-specific Abs against the infecting serotype. This could be due to the fact that the fraction enriched for sE-dimer specific antibodies is not homogeneous and still contains some remaining antibodies reacting to sE, lowering the neutralization potential of this fraction. However, anti-EDIII Abs exhibited significantly lower cross-neutralization potency against DENV1 compared to sE-dimer fraction-enriched Abs. This is in accordance with the identification and characterization of anti-EDIII mAbs, which are usually strong serotype-specific neutralizing antibodies (Ahmed et al, 2025).

It has been shown that FL epitopes are frequently exposed on immature or partially mature virions, making them accessible to sub-neutralizing Abs that promote Fcγ-mediated entry and viral replication in host cells (Rouvinski et al, 2017; Shukla et al, 2020). In addition, Fcγ-dependent uptake of immune complexes has been shown to inhibit type I interferon responses and promote a pro-inflammatory cytokine profile, leading to increased vascular permeability and impairment of innate and adaptive immune responses (Bournazos et al, 2020; Narayan and Tripathi, 2020; Ubol and Halstead, 2010). As type I interferon supports survival, clonal expansion and cytotoxicity of CD4+ and CD8 + T cell subsets, ADE-mediated infection could lead to suboptimal T cell responses. On the other hand, induction of protective CD4+ and CD8 + T cell responses early after infection could control ADE-mediated infection. Indeed, in previous studies, we observed that subclinical dengue cases are characterized by an expansion of CD4 + TEMRA cells, and a higher frequency of cytotoxic CD8 + EM (Gonnella et al, 2025). Understanding the interplay between antibody-mediated protection/enhancement and T cell-mediated protection represents an important avenue for future investigations.

Unlike anti-FL Abs, E-dimer-specific Abs bind quaternary epitopes present only on mature virions, avoiding non-neutralizing interactions with immature particles that are often associated with enhancement (Dejnirattisai et al, 2015; Rouvinski et al, 2015). Previous studies have demonstrated that mAbs targeting sE-dimer-dependent quaternary epitopes isolated from DENV-infected patients are cross-reactive with strong neutralizing capacity (Fibriansah et al, 2015; Young et al, 2020). The loss of the neutralizing potency but remaining ADE potency in the fraction of EDIII, sE and sE-dimer depleted Abs might be attributed to the presence and activity of anti-prM antibodies. Indeed, anti-prM Abs are well known to be poorly neutralizing but highly efficient at mediating ADE (Dejnirattisai et al, 2010).

Several limitations should be noted. First, we employed mouse mAb clone 4G2 to block the FL epitope; therefore, other FL-specific Abs that bind slightly different regions may not have been detected by our approach. Stabilization of the sE-dimer protein may also have altered binding sites for C10-like Abs, potentially affecting detection (Rouvinski et al, 2017). In addition, human antibodies with higher affinity for the FL epitope than 4G2 might compete for the FL binding site, leading to an underestimation of the anti-FL antibodies. However, binding of 1A5, a humanized anti-FL antibody, is abrogated in the presence of 4G2, confirming the validity of our strategy (Lay et al, 2025). Secondly, our functional characterization is based on standardized in vitro assays. In patients, ADE and neutralization potential of the antibodies will also depend on viremia of each individual patient, and antibody concentration and virus titers in the target tissues of the disease. Neutralization assays were performed using C6/36-derived DENV2, which is enriched for immature and partially mature virions; because virion maturation affects epitope accessibility, this may limit precise discrimination of antibody quality based on neutralization alone (Dejnirattisai et al, 2015; Maciejewski et al, 2020). Thirdly, the determination of immune history is poorly standardized across assays and laboratories. In our study, immune status was determined based on HI titers in the Cambodian cohort, and based on avidity ELISA in the Colombian cohort as per WHO guidelines (WHO Guidelines, 2009). We lack information on the previous infecting serotype of the patients and whether if patients underwent one or multiple prior DENV infections. Indeed, tertiary or quaternary dengue infection usually results in more mild disease, and more anti-sE antibodies are detected in individuals who experienced at least two prior DENV infections (Mpingabo et al, 2025; Olkowski et al, 2013). However, there is currently no method available to distinguish secondary from post-secondary infection in cross-sectional cohorts such as our cohort design. An alternative hypothesis, therefore, is that the observed antibody profiles may be a correlate of cumulative antigenic exposure rather than a direct determinant of disease severity. Specifically, it is plausible that individuals presenting with subclinical disease were disproportionately represented by those with a history of multiple prior infections. Consequently, the association between antibody fractions and disease severity could be influenced by immunological characteristics shaped by repeated exposure.

Strengths of the study include the evaluation of the antibody response during acute infection, and in individuals undergoing subclinical infection. Together with the functional characterization of the purified antibodies, we provide evidence in humans that FL antibodies determine disease susceptibility, while sE-dimer-specific antibodies protect from severe disease. Our findings determining the association between E protein epitope-specific antibodies and disease outcome complement studies using mAbs and animal models to demonstrate protective or pathogenic effects of the humoral immune response after DENV infection.

In conclusion, our findings emphasize the functional heterogeneity of anti-DENV2 E Ab subsets and the importance of epitope specificity in shaping clinical outcomes. We demonstrated that FL-targeting Abs may contribute to disease enhancement through ADE, while sE-dimer-specific Abs offer strong neutralization with minimal enhancement. These results provide important insights for future vaccine strategies, which may benefit from eliciting sE-dimer-specific responses while minimizing induction of enhancement-prone Abs such as those targeting the FL epitope. Investigating the kinetics and functions of E protein-specific Abs across DENV serotypes will also be essential to determine whether certain epitope-specific responses are universally protective or serotype-specific.

## Methods

**Table:** Reagents and tools table

Reagent/resource	Reference or source	Identifier or catalog number
**Experimental models**		
NA	NA	NA
NA	NA	NA
**Recombinant DNA**		
NA	NA	NA
NA	NA	NA
**Antibodies**		
mAb 1A5	GenScript Biotech	NA
4G2 (in-house production from hybridoma cell)	ATCC HB-112	NA
Commercial 4G2	GeneTex International Corp	GTX57154
(R-PE)-conjugated goat anti-human IgG	Fisher Scientific	109-115-098
**Oligonucleotides and other sequence-based reagents**		
NA	NA	NA
NA	NA	NA
**Chemicals, enzymes and other reagents**		
Tris (1 M)	Sigma	171321-01
Pierce IgG Elution Buffer	Thermo Scientific	21004
Fetal bovine serum	Gibco	16000-044
Penicillin-streptomycin	Gibco	15140-122
L-glutamine	Gibco	25030-024
Blasticidin	InvivoGen	Ant-bl-1
Leibovitz’s L-15 medium (L-15)	Sigma-Aldrich	L5520-500mL
Dulbecco’s modified Eagle medium (DMEM)	Sigma-Aldrich	D5796-500mL
Roswell Park Memorial Institute (RPMI)	Sigma-Aldrich	R0883-500ML
Polyethylene glycol	Sigma-Aldrich	P2139-500G
EDTA-trypsin	Gibco	15400-054
Zombie Aqua	BioLegend	423101
Fixation/permeabilization buffer set	BioLegend	424401
Permeabilization buffer	BioLegend	424401
Dulbecco’s Phosphate-Buffered Saline (DPBS) (10X), no calcium, no magnesium	Dominique	14200-067
Tween 20, 100% Nonionic Detergent	Bio-Rad	1706531
Bovine serum albumin (BSA)	Sigma-Aldrich	A4503-50G
Protein G Sepharose™ 4 Fas Flow	GE Healthcare	17-0618-01
Tryptose-phosphate broth	Gibco	T9157-500G
Commercial BioPlex Amine Coupling Kit	Bio-Rad	171406001
1-ethyl-3-(3-dimethyl-amino propyl carbodiimide hydrochloride (EDAC)	Bio-Rad	1530990
N-hydroxy-sulfosuccinimide sodium salt (S-NHS)	Thermo Fisher Scientific	24510
Biotin-Protein Ligase / BirA Enzyme	GeneCopoeia	BI001
BioPlex Seath Fluid	Bio-Rad	17000055
Quick Start Bradford 1x Dye Reagent	Bio-Rad	5000205
Protease inhibitor cocktail tablet	Roche	11697498001
**Software**		
GraphPad Prism	Version 10.3.1	
FlowJo	Version 10.8.1	
BioRender	License	
**Other**		
BioPlex pro magnetic COOH bead region 35	Bio-Rad	MC10035-01
BioPlex pro magnetic COOH bead region 36	Bio-Rad	MC10036-01
BioPlex pro magnetic COOH bead region 43	Bio-Rad	MC10043-01
Mini bio-spin chromatography columns	Bio-Rad	732-6207
Amicon Ultra-100 regenerated cellulose membrane	Sigma	UFC210024

### Ethics statement

The study was performed in accordance with the principles set out in the World Medical Association Declaration of Helsinki and the Department of Health and Human Services Belmont Report.

#### Cambodia cohort

Ethical approval for the clinical study was obtained from the National Ethics Committee for Health Research of Cambodia (NECHR number 2020-214). Written informed consent was obtained from all participants or from the legal guardians of participants under 16 years of age prior to inclusion in the study.

#### Colombia cohort

All work with human study participants was approved by the Stanford University Administrative Panel on Human Subjects in Medical Research (protocols 35460 and 50513) and by the ethics committees of the Fundación Valle del Lili (FVL; Cali, Colombia) and the Centro de Atención y Diagnóstico de Enfermedades Infecciosas (CDI; Bucaramanga, Colombia). Written informed consent was obtained from all adult participants, and from the parents or legal guardians, and assent was obtained from participants aged 2–17 years.

### Patient recruitment

#### Cambodia cohort

DENV cases were identified in the DENTHOM project from 2022 to 2024 among hospitalized patients presenting with acute dengue-like illness in Kampong Thom and Siem Reap provinces. Patients were recruited at Kampong Thom Provincial Hospital, Baray and Stuong district hospitals in Kampong Thom province and Jayavarman VII hospital in Siem Reap province. Blood samples were collected on the day of hospital admission (day 0, D0) and tested for DENV infection by quantitative reverse transcription polymerase chain reaction (RT-qPCR). Hospitalized patients with RT-qPCR-confirmed DENV infection were classified according to the WHO 1997 classification scheme into dengue fever (DF), dengue hemorrhagic fever (DHF), or dengue shock syndrome (DSS) (Paz-Bailey et al, 2024). To identify individuals with subclinical infections, a household investigation approach was used around RT-qPCR-confirmed index cases in Kampong Thom province. In laboratory-confirmed cases, clinical signs and symptoms were recorded retrospectively for 5 days and prospectively for 10 days, with follow-up visits on days 3, 5, and 10 (D3, D5, and D10) post-enrollment. Subclinical infection was defined as no or mild (<38 °C) fever for a maximum of 2 consecutive days and a maximum of 1 of the following symptoms for a maximum of 2 consecutive days: nausea or vomiting, rash, aches and pains. Individuals requiring medical care were excluded from subclinical cases. For all RT-qPCR-confirmed DENV cases, additional blood samples were collected on days 3, 10, and 60. Clinical and biological data were documented in case report forms. Samples from both hospitalized and subclinical cases were categorized by infection stage: acute phase (0–3 days after onset of symptoms), critical phase (4–6 days after onset of symptoms), early convalescence (7–14 days after onset of symptoms) and late convalescence (2 months post onset of symptoms). In the subclinical group who had no symptoms (*n* = 5 out of 13 individuals), D0 and D3 samples were categorized as acute and critical phases, respectively. Only patients undergoing a post-primary infection with DENV2 were included for analysis.

#### Colombia cohort

Study participants presented to the emergency room or clinics of FVL or CDI between March 2016 and January 2020. Enrollment criteria consisted of: (1) ≥2-year old; (2) presentation with an acute febrile illness (<7-day duration) associated with one or more of the following symptoms or signs: headache, rash, arthralgia, myalgia, retro-orbital pain, abdominal pain, positive tourniquet test, petechiae and bleeding; and (3) a positive result for dengue IgM antibody and/or NS1 antigen in a SD BIOLINE Dengue Duo combo device (Standard Diagnostic Inc.) test or clinical presentation highly consistent with dengue and subsequent confirmation of diagnosis via serological testing and RT-qPCR at Stanford. Patients were classified by infectious disease specialists—both on presentation and at recovery—as having dengue (D), dengue with warning signs (DWS) or severe dengue (SD) according to 2009 WHO criteria (WHO Guidelines, 2009). Patients who progressed to SD before enrollment were excluded from this study. The first day of fever (fever day 0) was defined by the patients or their relatives. Symptoms, warning signs and laboratory parameters (including complete blood count, chemistry and liver function test results) were documented by healthcare professionals. Whole venous blood samples (10–40 ml) were collected on enrollment, and PBMCs and serum samples were isolated. Sample transport, reception and processing were strictly controlled using personal data assistants with barcode scanners. For patients managed in an outpatient setting, follow up was conducted daily via phone calls (during which patients were provided information about the clinical warning signs and asked about their appearance) until full recovery when a final diagnosis was determined. Only patients undergoing a post-primary infection with DENV1 were included for analysis. Demographic data and clinical characteristics of dengue patients from both cohorts are described in Table EV1.

### Laboratory diagnosis

#### Cambodia cohort

Blood samples from hospitalized patients were tested for DENV and serotyped by RT-qPCR (Ou et al, 2020) at the Virology Unit of the *Institut Pasteur du Cambodge* (IPC). Only patients with confirmed DENV2 infection were included in the analysis. The distinction between primary and secondary DENV infections was made using the hemagglutination inhibition (HI) assay following WHO 2009 criteria using plasma samples obtained at D0 and D10 (WHO Guidelines, 2009). To minimize variability related to serotype or immune status, only patients undergoing secondary (post-primary) DENV2 infection were included in this study.

#### Colombia cohort

Serum samples were screened via a qualitative, single reaction, multiplex real-time reverse transcriptase PCR that detects Zika, chikungunya, and DENV RNA. To identify the specific DENV serotype and quantify the viral load, samples positive for DENV in the screening assay were serotyped and quantitated using a separate DENV multiplex RT-qPCR (Waggoner et al, 2013). To distinguish between primary and secondary infections, anti-DENV IgG was measured using the DENV Detect IgG ELISA kit (InBios) as per the manufacturer’s instructions. The plates were read at 450 nm using a Spectra Max M2 (Molecular Devices) system and the SoftMax pro 7.0.3 software. Presence or absence of DENV IgG was interpreted from the ratio of readings from DENRA and NCA wells without urea. A ratio of ≥2.84 was considered positive, 1.65–2.84 ‘equivocal’ and ≤1.65 as negative. Testing of the equivocal samples was repeated once. DENV IgG avidity was calculated by the ratio of the reading in the DENRA well without urea to the DENRA well with 8 M urea. For the samples with a positive result according to the DENRA/NCA ratio, high avidity (>0.6) was considered as secondary (post-primary) infection, and moderate (0.4–0.6) and low (<0.6) avidity as primary infection.

### Isolation of total IgG from human plasma

Total human IgG was isolated from confirmed dengue-positive plasma using affinity chromatography (Protein G sepharose 4 Fast Flow Resin, GE Healthcare), following the manufacturer’s instructions. Briefly, plasma samples were diluted with an equal volume of protease inhibitor (1 mM) (Roche) and incubated with pre-washed protein G sepharose beads in 1X phosphate-buffered saline (PBS, Thermo Scientific) on a rotator for 20 min at room temperature (RT). After incubation, the beads were washed with 1X PBS, and bound IgG was eluted using Pierce IgG Elution Buffer pH 2.8 (Thermo Scientific). The eluted fractions were immediately neutralized with 1 M Tris pH 8 (Sigma). IgG concentration was determined using the Bradford protein assay (Bio-Rad).

### DENV E protein epitope-specific IgG detection via microsphere-based immunoassay (MIA)

DENV E recombinant proteins, including EDIII (19 kDa), soluble E (sE) (50 kDa) and stabilized sE-dimer (100 kDa) proteins were produced, based on sequences of Cambodian DENV2 (accession number, AHG23166) or DENV1 isolate (accession number, AHG23167), in Drosophila S2 cells and two mutations (Leu107Cys and Ala313Cys) were introduced in the protein to construct two disulfide bonds which contain the sE in a dimeric form. A multiplex MIA was used to detect E protein epitope-specific Abs in human plasma samples, as previously described (Lay et al, 2025). For the DENV1-infected Colombian cohort, DENV1 recombinant proteins were used. For the DENV2-infected Cambodian cohort, DENV2 recombinant proteins were used. Briefly, recombinant DENV2 or DENV1 proteins (EDIII, soluble E (sE), and sE-dimer) were coupled to different color-coded magnetic microsphere beads (BioPlex Pro Magnetic COOH Beads, Bio-Rad) at optimized concentrations. Detection of DENV sE protein epitope-specific Abs was performed in separate wells. To block the FL epitope, mouse mAb clone 4G2 (GeneTex International Corp, Cat# GTX57154) was added to sE-coupled beads beforehand. After five washes, pre-diluted total IgG from human samples (100 µL at 1 µg/mL) or anti-DENV E mAbs (100 µL at 1 µg/mL) were added to wells containing beads coupled with EDIII, sE, sE-dimer, or 4G2-preblocked sE. The Ab-bead complexes were incubated for 1 h at RT in the dark on a plate shaker at 450 rpm. Subsequently, R-Phycoerythrin (R-PE)-conjugated goat anti-human IgG (AffiniPure, Fisher Scientific, Cat# 109-115-098) was added for detection, and fluorescence signals were measured using the BioPlex-200 system (Bio-Rad). The relative abundance of 4G2-like Abs (representing anti-FL Abs) was calculated by subtracting the median fluorescence intensity (MFI) of Abs bound to 4G2-blocked sE from the MFI of Abs bound to unblocked sE. Similarly, sE-dimer-specific Abs were quantified by subtracting the MFI of Abs bound to blocked sE from the MFI of Abs bound to the sE-dimer. The proportion of 4G2-like Abs within the total anti-sE Ab response was calculated by dividing the MFI of 4G2-like Abs by the MFI of total anti-sE Abs. The proportion of sE-dimer-specific Abs was calculated by dividing the MFI of sE-dimer-specific Abs by the MFI of the total sE-dimer-specific Abs.

### Purification of DENV2 E protein epitope-specific IgG from human plasma

Total IgG from 12 DENV2-infected individuals from the Cambodian cohort obtained during the critical phase was subjected to further purification. Samples were selected based on the following criteria in order to obtain sufficient amounts of purified antibodies for further downstream analysis: highest volume of plasma available (Cambodian cohort), patients with the highest anti-FL and anti-sE dimer antibody levels. DENV2 sE protein epitope-specific Ab subset were isolated using our previously established and validated protocol (Lay et al, 2025) (Fig. EV3). Briefly, C-terminally biotinylated DENV E proteins (EDIII, sE, and sE-dimer) were conjugated to streptavidin-resin beads according to the manufacturer’s instructions. The purified total IgG (200 µg) was first incubated with EDIII-coated beads on a rotator for 1 h at RT. The IgG-bead suspension was transferred to a gravity chromatography column (Bio-Rad) and centrifuged to remove unbound Abs. After washing, bound anti-EDIII Abs were eluted using Pierce IgG Elution Buffer (Thermo Scientific, pH 2.3) (fraction 1). The flow-through was then incubated with sE-dimer-coated beads for one hour at RT. After incubation and washing, the sE-dimer-specific Abs were eluted. The resulting flow-through was incubated with sE-coated beads to purify FL-specific Abs. The purity and epitope specificity of all Ab fractions were confirmed by MIA, and their concentrations were measured by in-house human total IgG ELISA.

### Virus and cell lines

Vero cells (ATCC CCL-81) were cultured in Dulbecco’s modified Eagle medium (DMEM; Sigma-Aldrich) supplemented with 5% fetal bovine serum (FBS, Gibco) and 100 U/mL penicillin-streptomycin (Gibco) at 37 °C in 5% CO_2_ atmosphere. The engineered U937-panFcγR expressing cells (expressing FcγRI, IIa, IIb, and III) (gift from Stylianos Bournazos, Rockefeller University) (Yamin et al, 2023), were maintained in Roswell Park Memorial Institute (RPMI) 1640 medium (Sigma-Aldrich) supplemented with 10% FBS, 1% L-glutamine (Gibco), 100 U/mL penicillin-streptomycin, and 10 µg/mL blasticidin (InvivoGen) at 37 °C in 5% CO_2_. *Aedes albopictus* C6/36 cells were cultured in Leibovitz’s L-15 medium (Sigma-Aldrich) supplemented with 10% FBS, 1% L-glutamine, 10% tryptose-phosphate broth (Gibco) and 100 U/mL penicillin-streptomycin at 28 °C. Cell lines were not authenticated. Cells were tested for mycoplasma contamination by PCR before execution of the experiments (Jean et al, 2017; Störmer et al, 2009)

DENV2 (New Guinea C strain, GenBank: AF038403) was propagated in C6/36 cells and concentrated using 40% polyethylene glycol (PEG) 8000 (Sigma-Aldrich). Virus titers were determined by focus-forming assay as previously described (Vo et al, 2022)

### Flow cytometry-based neutralization assay

Neutralization potential of sE protein epitope-specific Abs isolated from human plasma was evaluated using a flow cytometry-based assay as previously described (Roth et al, 2024). Due to the low amount of epitope-specific antibodies, experiments were performed in single. DENV2 neutralization was evaluated in 11 individuals, while cross-neutralization to DENV1 was only evaluated in five individuals due to the low amount of epitope-specific antibodies available. Briefly, Abs were serially diluted starting from 200 ng/mL in DMEM and incubated with DENV2 at a multiplicity of infection (MOI) of 1 for 1 h at 37 °C and 5% CO_2_. Virus-only controls (without Abs) were included. The Ab-virus complexes were transferred to 96-well culture plates containing Vero cells and incubated for 90 min. After washing, supplemented DMEM was added, and cells were further incubated for 24 h. Cells were detached with 2x EDTA-trypsin (Gibco), stained with Zombie Aqua viability dye (BioLegend), fixed, permeabilized and stained for DENV using anti-E protein Abs clone 4G2 (ATCC HB-112) conjugated with Alexa Fluor 488 (Invitrogen). Samples were analysed by flow cytometry (FACSCanto II, BD Biosciences). Dose-response neutralization curves were normalized to no-Ab controls and analysed by nonlinear regression with a variable slope constraining minimum and maximum responses to 0 and 100%, respectively (Graphpad Prism version 10.3.1)

### Antibody-dependent enhancement (ADE) Assay

ADE activity of isolated Abs was determined by using a cell-based assay adapted from our previous work (Vo et al, 2022). Instead of using wild-type U937 cells, U937-panFcγR expressing cells were used (Yamin et al, 2023). Due to the low amount of epitope-specific antibodies, experiments were performed in single. Briefly, serially diluted Abs were incubated with DENV2 at an MOI of 0.1 for one hour at 37 °C in 5% CO_2_. The humanized mAb 1A5 (GenScript Biotech), specific for the FL in EDII and based on a published sequence (Lai et al, 2008), was used as a positive control. Virus-only conditions served as negative controls. The Ab-virus complexes were transferred to 96-well U-bottom plates containing 8 × 10^4^ U937 cells per well and incubated for 90 min. After washing, cells were further incubated for 48 h. Cells were stained with Zombie Aqua viability dye, fixed, permeabilized and stained for intracellular DENV using anti-E protein Ab clone 4G2 (ATCC HB-112) conjugated with Alexa Fluor 488. Flow cytometry was used to determine the percentage of DENV-infected cells. Dose-dependent enhancement curves were generated, and the area under the curve (AUC) was calculated using GraphPad Prism (version 10.3.1).

### Statistical analysis

All statistical analyses and data visualizations were performed using GraphPad Prism (version 10.3.1). We performed the D’Agostino test to test for a Gaussian distribution. Since all data were not normally distributed, nonparametric tests, including the Mann–Whitney *U*- test, Friedman test, unpaired *t*-test, Dunn’s test, Bonferroni’s test, and paired Wilcoxon for multiple comparisons, were used as appropriate. A *p* value of <0.05 was considered statistically significant. Data were presented as median with interquartile range.

## Supplementary information

Table EV1

Peer Review File

Source data Fig. 1

Source data Fig. 2

Source data Fig. 3

Source data Fig. 4

Source data Fig. 5

Source data Fig. 6

Expanded View Figures

## Data Availability

This study includes no data deposited in external repositories. The source data of this paper are collected in the following database record: biostudies:S-SCDT-10_1038-S44321-026-00485-7.
