# Supplementary material for: Dengue virus envelope epitope-specific antibodies are associated with protection from severe dengue
Source: EMBO Mol Med. 2026 Aug 6;18(9):3448–73. doi: 10.1038/s44321-026-00485-7 (PMC13562538; doi:10.1038/s44321-026-00485-7)
Supplement: Supplementary file 1 [file 44321_2026_485_MOESM2_ESM.pdf]

EMBO Molecular Medicine

Dengue Virus Envelope epitope-specific antibodies are associated with protection from severe dengue

Sokchea Lay, Heidi Auerswald, Sievleang HENG, Sopheak Sorn, Sreymom Ken, Verónica Durán, David Esteban Rebellón Sanchez, Daniela Vinueza, Luis Angel Villar Centeno, Fernando Rosso, Shirit Einav, Anavaj Sakuntabhai, Felix Rey, Kevin Ariën, Sowath Ly, Veasna Duong, Giovanna Barba-Spaeth, and Tineke Cantaert

Corresponding authors: Tineke Cantaert (tineke.cantaert@pasteur.fr) , Heidi Auerswald (h.auerswald@izs.it)

Review Timeline:

Submission Date:	18th Mar 26
Editorial Decision:	8th Apr 26
Revision Received:	4th Jun 26
Editorial Decision:	24th Jun 26
Revision Received:	2nd Jul 26
Accepted:	2nd Jul 26

Editor: Zeljko Durdevic

Transaction Report:

(Note: With the exception of the correction of typographical or spelling errors that could be a source of ambiguity, letters and reports are not edited. Depending on transfer agreements, referee reports obtained elsewhere may or may not be included in this compilation. Referee reports are anonymous unless the Referee chooses to sign their reports.)

8th Apr 2026

Dear Dr. Cantaert,

Thank you for the submission of your manuscript to EMBO Molecular Medicine. We have now received feedback from the three reviewers who agreed to evaluate your manuscript. All three referees recognize interest of the study but also raise serious concerns that should be addressed in a major revision. If you would like to discuss further the points raised by the referees, I am available to do so via email or video. Let me know if you are interested in this option.

Further consideration of a revision that addresses reviewers' concerns in full will entail a second round of review. EMBO Molecular Medicine encourages a single round of revision only and therefore, acceptance or rejection of the manuscript will depend on the completeness of your responses included in the next, final version of the manuscript. For this reason, and to save you from any frustrations in the end, I would strongly advise against returning an incomplete revision.

We would welcome the submission of a revised version within three months for further consideration. Please let us know if you require longer to complete the revision.

I look forward to receiving your revised manuscript.

Yours sincerely,

Zeljko Durdevic

Zeljko Durdevic
Senior Editor
EMBO Molecular Medicine

*****

When preparing your revised manuscript, please refer to our guidelines: https://link.springer.com/journal/44321/submission-guidelines#cms-Revised-submissions. We perform an initial quality control of all revised manuscripts before re-review; failure to include requested items will delay the evaluation of your revision.

We require:

1) A .docx formatted version of the manuscript text (including legends for main figures, EV figures and tables). Please make sure that the changes are highlighted to be clearly visible.

2) Individual production quality figure files as .eps, .tif, .jpg (one file per figure). For guidance, download the 'Figure Guide PDF': https://media.springernature.com/original/springer-cms/rest/v1/content/27825798/data/v1.

3) A .docx formatted letter INCLUDING the reviewers' reports and your detailed point-by-point responses to their comments. As part of the EMBO Press transparent editorial process, the point-by-point response is part of the Review Process File (RPF), which will be published alongside your paper.

4) A complete author checklist, which you can download from our author guidelines. Please insert information in the checklist that is also reflected in the manuscript. The completed author checklist will also be part of the RPF.

5) Please note that all corresponding authors are required to supply an ORCID ID for their name upon submission of a revised manuscript.

6) It is mandatory to include a 'Data Availability' section after the Materials and Methods. Before submitting your revision, primary datasets produced in this study need to be deposited in an appropriate public database, and the accession numbers and database listed under 'Data Availability'. Please remember to provide a reviewer password if the datasets are not yet public.

In case you have no data that requires deposition in a public database, please state so in this section. Note that the Data Availability Section is restricted to new primary data that are part of this study.

7) For data quantification: please specify the name of the statistical test used to generate error bars and P values, the number (n) of independent experiments (specify technical or biological replicates) underlying each data point and the test used to calculate p-values in each figure legend. The figure legends should contain a basic description of n, P and the test applied. Graphs must include a description of the bars and the error bars (s.d., s.e.m.).

8) At EMBO Press we ask authors to provide source data for the main manuscript figures. You will receive a separate email with instructions for providing source data with your revised manuscript, including how to upload and organize the files.

9) Our journal encourages inclusion of *data citations in the reference list* to directly cite datasets that were re-used and obtained from public databases. Data citations in the article text are distinct from normal bibliographical citations and should directly link to the database records from which the data can be accessed. In the main text, data citations are formatted as follows: "Data ref: Smith et al, 2001" or "Data ref: NCBI Sequence Read Archive PRJNA342805, 2017". In the Reference list, data citations must be labeled with "[DATASET]". A data reference must provide the database name, accession number/identifiers and a resolvable link to the landing page from which the data can be accessed at the end of the reference.

10) We replaced Supplementary Information with Expanded View (EV) Figures and Tables that are collapsible/expandable online. A maximum of 5 EV Figures can be typeset. EV Figures should be cited as 'Figure EV1, Figure EV2" etc... in the text and their respective legends should be included in the main text after the legends of regular figures.

- For the figures that you do NOT wish to display as Expanded View figures, they should be bundled together with their legends in a single PDF file called *Appendix*, which should start with a short Table of Content. Appendix figures should be referred to in the main text as: "Appendix Figure S1, Appendix Figure S2" etc.

- Additional Tables/Datasets should be labeled and referred to as Table EV1, Dataset EV1, etc. Legends have to be provided in a separate tab in case of .xls files. Alternatively, the legend can be supplied as a separate text file (README) and zipped together with the Table/Dataset file.

11) The paper explained: EMBO Molecular Medicine articles are accompanied by a summary of the articles to emphasize the major findings in the paper and their medical implications for the non-specialist reader. Please provide a draft summary of your article highlighting

- the medical issue you are addressing,

- the results obtained and

- their clinical impact.

This may be edited to ensure that readers understand the significance and context of the research. Please refer to any of our published articles for an example.

12) Author contributions: You will be asked to provide CRediT (Contributor Role Taxonomy) terms in the submission system. These replace a narrative author contribution section in the manuscript.

13) A Conflict of Interest statement should be provided in the main text.

14) Every published paper includes a 'Synopsis' to further enhance discoverability. Synopses are displayed on the journal webpage and are freely accessible to all readers. They include a short stand first (maximum of 300 characters, including space) as well as 2-5 one-sentences bullet points that summarizes the paper. Please write the bullet points to summarize the key NEW findings. They should be designed to be complementary to the abstract - i.e. not repeat the same text. We encourage inclusion of key acronyms and quantitative information (maximum of 30 words / bullet point). Please use the passive voice. Please attach these in a separate file or send them by email, we will incorporate them accordingly.

Please also suggest a striking image or visual abstract to illustrate your article as a PNG file 550 px wide x 300-600 px high.

15) Include a Reagents and Tools Table as part of the Methods section, which can be downloaded from our author guidelines.

EMBO Molecular Medicine has a "scooping protection" policy, whereby similar findings that are published by others during review or revision are not a criterion for rejection. Should you decide to submit a revised version, I do ask that you get in touch after three months if you have not completed it, to update us on the status.

***** Reviewer's comments *****

Referee #1 (Comments on Novelty/Model System for Author):

I do not find any major or minor issue with this manuscript, the model systems used or applied statistics.

Referee #1 (Remarks for Author):

This is a very straight forward and highly relevant study exploring the type of protective vs ADE conferring antibody response in the course of secondary DENV infection, that poses a high risk of severe dengue. By comprehensive antibody profiling of patient samples from an adequate cohort the authors find that FL-targeting abs (4G2-like) are highly increased during the critical phase of infection versus those that target the quarterny E-domain. This sE-dimer Abs were enriched in classical dengue fever patients, as compared to severe dengue. From this correlation they hypothesize that 4G2-like abs might be less neutralizing while promoting ADE and sE-dimer could be potent neutralizers with less ADE activity. This hypothesis was also proven experimentally.
Altogether, I would like to congratulate the authors for a very well conducted and relevant study. The manuscript is written clearly and understandably and I have nearly no critique to the data and the overall presentation, interpretation or methodology.
Minor thing is that the MFI IgG in Figure 2 A comparing sE to total sE-dimer looks fairly similar and it is hard to believe that the p-value difference is 0.0005 (even though these are quite some samples).
Figure 5 lowers my enthusiasm for the study a little bit, since I would have expected a final set of data that assesses cross neutralization of the diffrent ab classes as another essenial mechanism to protect from severe dengue. Along these lines, I would have expected that sE-dimer Abs are better cross neutralizers that 4G2-like abs. Please repeat the neutralization assay with DENV1.

Referee #2 (Comments on Novelty/Model System for Author):

Longstanding question with significant relevance to the field. Some limitations in terms of how the data are presented and interpreted, but which could be improved.

Referee #2 (Remarks for Author):

In this manuscript, Lay et al dissect dengue virus (DENV) epitope specific antibody responses in serum samples collected from volunteers experiencing symptomatic, symptomatic, or severe post-primary DENV-2 infections. This study seeks to fill a longstanding knowledge gap in the field regarding the relationship between the abundance of IgG antibodies with discrete epitope specificities and the clinical severity of the DENV infection. The authors leverage a powerful analysis modality and incredibly well-designed cohort.

1) The authors position many of the observations made in the study through the lens of ADE, seeking to link the clinical outcome of DENV-2 infection with the abundance of infection-enhancing/FL-specific IgG antibodies. However, the window where a significant bias towards 4G2-like antibodies is observed/described was the critical phase of DENV infection (4-6 days after fever onset). This seems like an odd timepoint to analyze, as viremia has generally resolved by this point and the anamnestic response to infection has begun, muddying the pre-infection antibody profile. It seems that a more logical time point to perform this analysis would be the earliest sample available, where the antibody response to the newly infecting serotype is minimal and there is still virus in circulation (and able to be enhanced).
2) It's somewhat unclear how the time points were aligned between patients experiencing symptomatic and subclinical infections. In the methods section it states that subclinical infections were defined and no or mild fever, but timing of sample collection (acute, critical phase, early convalescence, and late convalescence) is all defined relative to the day of fever onset. What fraction of "subclinical" infections did not result in a fever, and how were collection time points assigned for these volunteers?
3) The use of DENV-2 antigens is well justified for the Cambodia cohort, as volunteers were selected because they were experiencing a post-primary DENV-2 infection. However, all the volunteers in the Colombian cohort experienced a DENV-1 infection. Given the depletion and analysis strategy, can the authors comment on how this may impact the interpretation of the data shown in Figure S2?
4) It might simplify interpretation if Figure 3A and 3B were combined into a single panel, comparing subclinical vs DF vs DHF. Splitting first by subclinical and hospitalized and then between DF and DHF is a little jarring

Minor points:
1) Throughout the manuscript and figure it would be helpful if the antigens used were described as "DENV-2" rather than "DENV". The use of DENV-2 antigens only is well justified given the samples utilized, but it would significantly help improve

clarity to have this explicitly stated in both the figures and text.
2) In figure 4, it would be helpful to have a heading for each figure indicating the source of the antibody, somewhat like what is shown in Fig S4

Referee #3 (Remarks for Author):

Lay and co-authors present a paper where they longitudinally profile antibody responses against the DENV E-protein in a cohort of Cambodian children with subclinical secondary infection (n = 19), DF (n = 25) and DHF/DSS (n= 26) during the acute (day 0-3 post fever onset), critical (day 4-6), early convalescence (day 7-14) and late convalescence (2 months) phases of illness. All individuals had post-primary/secondary DENV-2 infection. They report that a higher proportion of sE-dimer specific Abs were associated with a lower risk of severe dengue, while conversely a higher proportion of Fusion loop-binding Abs were associated with an increased risk of hospitalized dengue. They further validate their findings in a separate cohort. In addition, they report higher neutralization potential of sE-dimer specific Abs and evidence of in vitro ADE by fusion-loop binding Abs.

Recruiting and performing longitudinal sampling of such a cohort, which includes both subclinical and clinical dengue, across the various phases of acute and convalescent infection is complex and not without challenges, and I commend the authors on this effort. However, I do have some comments, in particular relating to the interpretation of the findings based on the data presented.

Major comments:
1. The authors state that all individuals in the Cambodian cohort were experiencing a secondary heterologous DENV-2 infection, based on high DENV IgG avidity. However, this would not distinguish between secondary and tertiary infections. Indeed, as the authors themselves note, anti-EDE antibodies are predominantly observed following post-secondary rather than post-primary infections (Dejnirattisai et al, Nat Immunol 2015; Mpingabo et al, Sci Transl Med 2025). Thus, there is a substantial possibility that individuals with milder or subclinical disease were in fact experiencing tertiary, rather than secondary, DENV infections. Although the authors acknowledge this as a potential limitation, this has important implications for the interpretation of their findings, specifically the conclusion that sE-dimer-specific antibodies are protective in secondary DENV infection. If a proportion of these individuals were instead undergoing tertiary infection, the observed association may reflect immune profiles characteristic of higher order exposures rather than true correlates of protection during secondary infection.

2. Following on from the previous point, Figures 1F and 2B show that a subset of individuals already have relatively high levels of sE-dimer-specific antibodies during the very early acute phase (day 0-3). Who were these individuals? Specifically, do these participants predominantly belong to the subclinical group? If so, this observation may further support the possibility that some of these individuals had experienced prior multiple DENV exposures, which could influence the interpretation of the relationship between sE-dimer-specific antibodies and protection from severe secondary DENV infection.

3. For comparisons of the proportions of different antibody types across patients with differing clinical outcomes, the authors selected the critical phase time point (day 4-6), stating that "highest Ab titers are observed during the critical phase, where severe symptoms are likely to occur." However, a counterargument is that by this stage of illness, hyperinflammation may already be established, potentially limiting the capacity of antibodies to exert a protective effect. Indeed, the authors have previously reported that plasmablast expansion occurs during the late inflammatory stage of DENV infection (Gonella et al, Cell Host & Microbe 2025), whereas earlier functional T cell responses appear to be associated with protection. This raises the question of whether antibody measurements at this relatively late time point can meaningfully inform mechanisms of protection.

4. Table 1. RNAemia levels of the 3 groups seem to be similar despite the differing clinical severity. Given that higher viremia levels are known to correlate with dengue disease severity, can the authors comment on this? Could it be due to differences in sampling time-points between the 3 groups (again this would affect the interpretability of the Ab findings).

Minor comments:
1. The methodology/timepoints for participant enrolment and sampling are quite
complex, especially for the Cambodian cohort. It may be helpful to have a graphic of figure to explain this.
2. There is an error in Figure 2. The first column of the x-axis should be "EDIII" and not "EDII".
3. In a number of instances, the authors refer to antibody "titers" when describing their results. This terminology is somewhat inaccurate as only MFI is reported - serial dilutions assays to determine actual Ab titers were not performed.

Referee #1 (Remarks for Author):

This is a very straight forward and highly relevant study exploring the type of protective vs ADE conferring antibody response in the course of secondary DENV infection, that poses a high risk of severe dengue. By comprehensive antibody profiling of patient samples from an adequate cohort the authors find that FL-targeting abs (4G2-like) are highly increased during the critical phase of infection versus those that target the quaternary E-domain. This sE-dimer Abs were enriched in classical dengue fever patients, as compared to severe dengue. From this correlation they hypothesize that 4G2-like abs might be less neutralizing while promoting ADE and sE-dimer could be potent neutralizers with less ADE activity. This hypothesis was also proven experimentally.
Altogether, I would like to congratulate the authors for a very well conducted and relevant study. The manuscript is written clearly and understandably and I have nearly no critique to the data and the overall presentation, interpretation, or methodology.
Minor thing is that the MFI IgG in Figure 2 A comparing sE to total sE-dimer looks fairly similar and it is hard to believe that the p-value difference is 0.0005 (even though these are quite some samples).
Figure 5 lowers my enthusiasm for the study a little bit, since I would have expected a final set of data that assesses cross neutralization of the different ab classes as another essential mechanism to protect from severe dengue. Along these lines, I would have expected that sE-dimer Abs are better cross neutralizers that 4G2-like abs. Please repeat the neutralization assay with DENV1.

We would like to thank referee 1 for the careful evaluation of the manuscript and for the congratulations on our well conducted and relevant study. We agree that adding information on cross-neutralization is a valuable addition to the manuscript, and we would like to thank the reviewer for this suggestion. As we are working with small sample volumes, the obtained amount of epitope-specific antibodies is limited to perform functional assays. However, from 5 individuals, we have obtained sufficient antibodies of all 3 subsets (sE-dimer specific, FL, EDIII) to perform additional cross-neutralization assays with DENV1. Indeed, antibodies targeting quaternary epitopes have been reported to be potentially cross-neutralizing. In line with these findings originating from mAbs, sE-dimer specific enriched Abs exhibited significantly higher cross-neutralizing potency against DENV1 compared to anti-FL antibodies, confirming our hypothesis. Interestingly, anti-EDIII Abs exhibited significantly lower cross-neutralization potency against DENV1 compared to sE-dimer fraction enriched Abs. This is in accordance with the identification and characterization of anti-EDIII mAbs, which are usually strong serotype-

specific neutralizing antibodies. These data have now been added to the manuscript as **Figure 6** (line 390-398, line 449-450, line 459-462).

We have double checked the statistical analysis of the MFI IgG in Figure 2A comparing sE to total sE-dimer, and the p value and analysis was correct.

Referee #2 (Comments on Novelty/Model System for Author):

Longstanding question with significant relevance to the field. Some limitations in terms of how the data are presented and interpreted, but which could be improved.

Referee #2 (Remarks for Author):

In this manuscript, Lay et al dissect dengue virus (DENV) epitope specific antibody responses in serum samples collected from volunteers experiencing symptomatic, symptomatic, or severe post-primary DENV-2 infections. This study seeks to fill a longstanding knowledge gap in the field regarding the relationship between the abundance of IgG antibodies with discrete epitope specificities and the clinical severity of the DENV infection. The authors leverage a powerful analysis modality and incredibly well-designed cohort.

We would like to thank referee 2 for their time allocated to conduct a careful evaluation of our manuscript.

1) The authors position many of the observations made in the study through the lens of ADE, seeking to link the clinical outcome of DENV-2 infection with the abundance of infection-enhancing/FL-specific IgG antibodies. However, the window where a significant bias towards 4G2-like antibodies is observed/described was the critical phase of DENV infection (4-6 days after fever onset). This seems like an odd timepoint to analyze, as viremia has generally resolved by this point and the anamnestic response to infection has begun, muddying the pre-infection antibody profile. It seems that a more logical time point to perform this analysis would be the earliest sample available, where the antibody response to the newly infecting serotype is minimal and there is still virus in circulation (and able to be enhanced).

We thank the reviewer for the comment. We used the critical phase timepoint as this is the time where severe disease might occur, and this is the timepoint where the antibody titers are the highest, indeed, reflecting a mix of both pre-existing and newly generated antibodies. At this time point, viral load is decreasing in the blood, but still measurable. For example, this is shown in a study of 2340 Vietnamese patients recently (Vuong et al, Elife, 2024, PMID: 38904662, see figure 2, 3). Hence, this timepoint was chosen. However, we agree with the reviewer that

evaluating the proportion of E epitope specific antibodies at the acute phase is of interest. In the cohort from Columbia, plasma samples at 2-7 days post-onset were included, thereby presenting a mix of the acute and critical timepoint, as we only had access to one timepoint (**Figure S2**). In the Cambodian cohort, which constituted a longitudinal follow up during the course of the infection, we now included the comparison of epitope-specific antibodies during the acute phase of infection (**Figure 3A-B)**.

2) It's somewhat unclear how the time points were aligned between patients experiencing symptomatic and subclinical infections. In the methods section it states that subclinical infections were defined and no or mild fever, but timing of sample collection (acute, critical phase, early convalescence, and late convalescence) is all defined relative to the day of fever onset. What fraction of "subclinical" infections did not result in a fever, and how were collection time points assigned for these volunteers?

We thank the reviewer for this comment. Line 146-147 (methods) and line 308-309 (results) in the original manuscript already stated: *In the subclinical group who had no symptoms, D0 and D3 samples were categorized as acute and critical phases, respectively*. Five out of 13 individuals did not show any symptoms. We have now added this information to that sentence. In addition, we clarified that the alignment was based on onset of symptoms (not fever), as some subclinical individuals had symptoms but no fever (see line 145-147 of the new version). So now, the phases are defined as follows: Acute phase (0-3 days after onset of symptoms), Critical phase (4-6 days after onset of symptoms), Early recovery phase (7-14 days after onset of symptoms), Recovery phase (2 months post onset of symptoms).

3) The use of DENV-2 antigens is well justified for the Cambodia cohort, as volunteers were selected because they were experiencing a post-primary DENV-2 infection. However, all the volunteers in the Colombian cohort experienced a DENV-1 infection. Given the depletion and analysis strategy, can the authors comment on how this may impact the interpretation of the data shown in Figure S2?

We thank the reviewers for the comment. The results in figure S2 were obtained using DENV1 derived antigens, this was briefly mentioned in the figure legend of the original manuscript (*Anti-DENV1 antibody (Ab) subsets were measured using microsphere-based immunoassay (MIA).* We further clarified this now and have added to the figure legend: *Anti-DENV1 antibody (Ab) subsets were measured using microsphere-based immunoassay (MIA) using DENV1 derived sE, sE-dimer and EDIII recombinant proteins.* In addition, we clarified this in the relevant results section as well: "*To validate these findings, we analysed the epitope-specific Ab profiles in an independent cohort undergoing a RT-PCR confirmed secondary DENV1 infection from Colombia*

using DENV1-derived recombinant E proteins." We noted this information was absent from the materials and methods and have now updated that section. We apologize for this unclarity in the previous version of the manuscript.

4) It might simplify interpretation if Figure 3A and 3B were combined into a single panel, comparing subclinical vs DF vs DHF. Splitting first by subclinical and hospitalized and then between DF and DHF is a little jarring.

We thank the reviewer for the comment, however, both comparisons answer different research questions: are there differences in epitope-specific antibodies associated to disease susceptibility (Figure 3A) and/or to disease severity (Figure 3B)? Therefore, we would like to retain the figure as presented.

Minor points:

1) Throughout the manuscript and figure it would be helpful if the antigens used were described as "DENV-2" rather than "DENV". The use of DENV-2 antigens only is well justified given the samples utilized, but it would significantly help improve clarity to have this explicitly stated in both the figures and text.

We agree with the reviewer and have modified this throughout the text, figures and figure legends.

2) In figure 4, it would be helpful to have a heading for each figure indicating the source of the antibody, somewhat like what is shown in Fig S4

We agree with the reviewer and have added the antibody source on top of the panels for clarity.

Referee #3 (Remarks for Author):

Lay and co-authors present a paper where they longitudinally profile antibody responses against the DENV E-protein in a cohort of Cambodian children with subclinical secondary infection (n = 19), DF (n = 25) and DHF/DSS (n= 26) during the acute (day 0-3 post fever onset), critical (day 4-6), early convalescence (day 7-14) and late convalescence (2 months) phases of illness. All individuals had post-primary/secondary DENV-2 infection. They report that a higher proportion of sE-dimer specific Abs were associated with a lower risk of severe dengue, while conversely a higher proportion of Fusion loop-binding Abs were associated with an increased risk of hospitalized dengue. They further validate their findings in a separate cohort. In addition, they report higher neutralization potential of sE-dimer specific Abs and evidence of in vitro ADE by fusion-loop binding Abs.

Recruiting and performing longitudinal sampling of such a cohort, which includes both subclinical and clinical dengue, across the various phases of acute and convalescent infection is complex and not without challenges, and I commend the authors on this effort. However, I do have some comments, in particular relating to the interpretation of the findings based on the data presented.

We would like to thank referee 3 for the careful evaluation of the manuscript and for their appreciation of the complexity of the cohort recruitment.

Major comments:

1. The authors state that all individuals in the Cambodian cohort were experiencing a secondary heterologous DENV-2 infection, based on high DENV IgG avidity. However, this would not distinguish between secondary and tertiary infections. Indeed, as the authors themselves note, anti-EDE antibodies are predominantly observed following post-secondary rather than post-primary infections (Dejnirattisai et al, Nat Immunol 2015; Mpingabo et al, Sci Transl Med 2025). Thus, there is a substantial possibility that individuals with milder or subclinical disease were in fact experiencing tertiary, rather than secondary, DENV infections. Although the authors acknowledge this as a potential limitation, this has important implications for the interpretation of their findings, specifically the conclusion that sE-dimer-specific antibodies are protective in secondary DENV infection. If a proportion of these individuals were instead undergoing tertiary infection, the observed association may reflect immune profiles characteristic of higher order exposures rather than true correlates of protection during secondary infection.

We thank the reviewer for raising this point. We agree with this comment and have indeed already mentioned this in the limitation section of our manuscript. We have adapted now

throughout the manuscript the notion “post-primary infection” rather than “secondary infection” to reflect this limitation.

We agree that it could be possible that subclinical disease might be due to a higher order infection. This alternative hypothesis has been added to the manuscript (line 505-510). “*An alternative hypothesis, therefore, is that the observed antibody profiles may be a correlate of cumulative antigenic exposure rather than a direct determinant of disease severity. Specifically, it is plausible that individuals presenting with subclinical disease were disproportionately represented by those with a history of multiple prior infections. Consequently, the association between antibody fractions and disease severity could be influenced by immunological characteristics shaped by repeated exposure.*

2. Following on from the previous point, Figures 1F and 2B show that a subset of individuals already have relatively high levels of sE-dimer-specific antibodies during the very early acute phase (day 0-3). Who were these individuals? Specifically, do these participants predominantly belong to the subclinical group? If so, this observation may further support the possibility that some of these individuals had experienced prior multiple DENV exposures, which could influence the interpretation of the relationship between sE-dimer-specific antibodies and protection from severe secondary DENV infection.

We thank the reviewer for this question. In response to this question and the question below, we have now added the comparative analysis of the proportions of epitope-specific antibodies during the acute phase of infection (**Figure 3 A, B**). A similar percentage of both subclinical and hospitalized patients had detectable levels of sE-dimer specific antibodies already in the acute phase of infection (6/13; 46% in the subclinical group versus 13/33; 39% in the hospitalized group). These data have now been added to the manuscript (line 334-337)

3. For comparisons of the proportions of different antibody types across patients with differing clinical outcomes, the authors selected the critical phase time point (day 4-6), stating that "highest Ab titers are observed during the critical phase, where severe symptoms are likely to occur." However, a counterargument is that by this stage of illness, hyperinflammation may already be established, potentially limiting the capacity of antibodies to exert a protective effect. Indeed, the authors have previously reported that plasmablast expansion occurs during the late inflammatory stage of DENV infection (Gonella et al, Cell Host & Microbe 2025), whereas earlier functional T cell responses appear to be associated with protection. This raises the question of whether antibody measurements at this relatively late time point can meaningfully inform mechanisms of protection.

We thank the reviewer for this comment. We used the critical phase timepoint as this is the time where severe disease might occur, and this is the timepoint where the antibody titers are the highest, indeed, reflecting a mix of both pre-existing and newly generated antibodies. At this time point, viral load is decreasing in the blood, but still measurable. For example, this is shown in a study of 2340 Vietnamese patients recently (Vuong et al, Elife, 2024, PMID: 38904662, figure 2, 3). Hence, this timepoint was chosen. Indeed, in our manuscript by Gonnella et al, Cell Host & Microbe 2025, we included patients in the acute phase of infection, 0-4 days after onset of symptoms.

We agree that investigating earlier timepoints could be informative, and have now added these data to the manuscript (**Figure 3A, B**). Due to the low number of patients with a positive titer, the observed differences are not significant.

4. Table 1. RNAemia levels of the 3 groups seem to be similar despite the differing clinical severity. Given that higher viremia levels are known to correlate with dengue disease severity, can the authors comment on this? Could it be due to differences in sampling time-points between the 3 groups (again this would affect the interpretability of the Ab findings).

We thank the reviewer for this comment. Over all our studies including subclinical patients, we have not been able to observe differences in RNAemia (PMID: 28855396, PMID: 40580952). This could be due to the small sample size of our cohorts, as these include subclinical cases and are powered for immunological analysis, and the fact that we evaluate RNAemia within 72 of onset of symptoms. Indeed, for example, an association between RNAemia and disease severity was observed in a cohort of 2340 Vietnamese patients from day 6 onwards (Vuong et al, Elife, 2024, PMID: 38904662).

Minor comments:

1. The methodology/timepoints for participant enrolment and sampling are quite complex, especially for the Cambodian cohort. It may be helpful to have a graphic of figure to explain this.

We thank the reviewer for this constructive remark, and have added this now in **Figure 1B.**

2. There is an error in Figure 2. The first column of the x-axis should be "EDIII" and not "EDII".

We have corrected this.

3. In a number of instances, the authors refer to antibody "titers" when describing their results.

This terminology is somewhat inaccurate as only MFI is reported - serial dilutions assays to determine actual Ab titers were not performed.

We agree and have changed the term “titers” to “amount” where appropriate.

24th Jun 2026

Dear Dr. Cantaert,

Thank you for submitting your revised manuscript to EMBO Molecular Medicine. I am pleased to inform you that we will be able to accept your manuscript pending the following final amendments:

1) Figures: Please upload main and EV figures as high-resolution files in TIFF, EPS or PDF format. Main figure files should be renamed to Figure 1 etc. and EV figure files should be Figure EV1 etc.
2) Source Data: Please upload source data for the main figures as one zipped file per figure.
3) In the main manuscript file, please do the following:
- Please address all comments suggested by our data editors listed below:
o Figure legends:
1. Please note that the exact p values are not provided in the legends of 1D-G; 2A,C,E,G; 3D; 4C,D; EV-1A-D.
2. Please indicate the statistical test used for data analysis in the legends of figures 1C-G; EV-1A-D.
3. Please note that information related to n is missing in the legends 4A-D; EV-5F.
- The manuscript sections should be in the following order: Title page - Abstract - Introduction - Results - Discussion - Methods - Data Availability - Acknowledgments - Disclosure Statement & Competing Interests - References - Figure Legends - (Main Tables with legends if applicable) - Expanded View Figure Legends.
- Rename Material and Methods to Methods.
- In Methods, provide the statement that the experiments involving human subjects conformed to the principles set out in the WMA Declaration of Helsinki and the Department of Health and Human Services Belmont Report.
- Indicate in legends number and nature of replicates and exact p= values, not a range, along with the statistical test used. To keep the figures "clear" some authors found providing an Appendix table Sx with all exact p-values preferable. You are welcome to do this if you want to.
- Please rename "conflict of interest" to "Disclosure and competing interests statement". We updated our journal's competing interests policy in January 2022 and request authors to consider both actual and perceived competing interests. Please review the policy https://www.embopress.org/competing-interests and update your competing interests if necessary.
- Author contributions: Please remove it from the manuscript and specify author contributions in our submission system. CRediT has replaced the traditional author contributions section because it offers a systematic machine-readable author contributions format that allows for more effective research assessment. You are encouraged to use the free text boxes beneath each contributing author's name to add specific details on the author's contribution. More information is available in our guide to authors:
https://www.embopress.org/page/journal/17574684/authorguide#authorshipguidelines
- Correct the reference citation in the text and reference list. In the text a reference should be cited by author and year of publication. Include a space between a word and the opening parenthesis of the reference that follows. In the reference list, citations should be listed in alphabetical order. Where there are more than 10 authors on a paper, 10 will be listed, followed by "et al.". DOIs should only be used for preprints and datasets that have not been published yet. Please check "Author Guidelines" for more information.
https://www.embopress.org/page/journal/17574684/authorguide#referencesformat
- In data availability statement please remove provided text and add the sentence "This study includes no data deposited in external repositories."
4) Funding: Make sure that information about all sources of funding are complete in both our submission system and in the manuscript. All funders and their grants need to be added to the system as separate entries. Please merge "Funding" with "Acknowledgments".
5) Tables:
- Please upload Table EV1 with its legend as a separate file.
- Please upload Table EV2 as Reagents and Tools Table. More information on how to adhere to this format as well as downloadable templates (.docx) for the Reagents and Tools Table can be found in our author guidelines:
https://www.embopress.org/page/journal/17574684/authorguide#structuredmethods
6) The Paper Explained: Please add it to the main manuscript file.
7) Synopsis:
- Synopsis image: Please provide a visual abstract as a high-resolution .jpeg file exactly 550 px-wide x 300 pixels high to illustrate your article.
- Please check your synopsis text and image before submission with your revised manuscript. Please be aware that in the proof stage minor corrections only are allowed (e.g., typos).
8) As part of the EMBO Publications transparent editorial process (see our Editorial at http://embomolmed.embopress.org/content/2/9/329), EMBO Molecular Medicine will publish online a Review Process File (RPF) to accompany accepted manuscripts. This file will be published in conjunction with your paper and will include the anonymous referee reports, your point-by-point response and all pertinent correspondence relating to the manuscript. Let us know if you want to remove or not any figures from it prior to publication. Please note that the Authors checklist will be published at the end of the RPF.

9) Please provide a point-by-point letter INCLUDING my comments as well as the reviewer's reports and your detailed responses (as Word file).

I look forward to reading a new revised version of your manuscript as soon as possible.

Yours sincerely,

Zeljko Durdevic

Zeljko Durdevic
Senior Editor
EMBO Molecular Medicine

*** Instructions to submit your revised manuscript ***

*** PLEASE NOTE *** As part of the EMBO Publications transparent editorial process initiative (see our Editorial at https://www.embopress.org/doi/pdf/10.1002/emmm.201000094), EMBO Molecular Medicine will publish online a Review Process File to accompany accepted manuscripts.

In the event of acceptance, this file will be published in conjunction with your paper and will include the anonymous referee reports, your point-by-point response and all pertinent correspondence relating to the manuscript. If you do NOT want this file to be published, please inform the editorial office at contact@embomolmed.org.

*****

When preparing your revised manuscript, please refer to our guidelines: https://link.springer.com/journal/44321/submission-guidelines#cms-Revised-submissions. We perform an initial quality control of all revised manuscripts before re-review; failure to include requested items will delay the evaluation of your revision.

We require:

1) A .docx formatted version of the manuscript text (including legends for main figures, EV figures and tables). Please make sure that the changes are highlighted to be clearly visible.

2) Individual production quality figure files as .eps, .tif, .jpg (one file per figure). For guidance, download the 'Figure Guide PDF': https://media.springernature.com/original/springer-cms/rest/v1/content/27825798/data/v1.

3) A .docx formatted letter INCLUDING the reviewers' reports and your detailed point-by-point responses to their comments. As part of the EMBO Press transparent editorial process, the point-by-point response is part of the Review Process File (RPF), which will be published alongside your paper.

4) A complete author checklist, which you can download from our author guidelines. Please insert information in the checklist that is also reflected in the manuscript. The completed author checklist will also be part of the RPF.

5) Please note that all corresponding authors are required to supply an ORCID ID for their name upon submission of a revised manuscript.

6) It is mandatory to include a 'Data Availability' section after the Materials and Methods. Before submitting your revision, primary datasets produced in this study need to be deposited in an appropriate public database, and the accession numbers and database listed under 'Data Availability'. Please remember to provide a reviewer password if the datasets are not yet public.

In case you have no data that requires deposition in a public database, please state so in this section. Note that the Data Availability Section is restricted to new primary data that are part of this study.

7) For data quantification: please specify the name of the statistical test used to generate error bars and P values, the number (n) of independent experiments (specify technical or biological replicates) underlying each data point and the test used to calculate p-values in each figure legend. The figure legends should contain a basic description of n, P and the test applied. Graphs must include a description of the bars and the error bars (s.d., s.e.m.).

8) At EMBO Press we ask authors to provide source data for the main manuscript figures. You will receive a separate email with instructions for providing source data with your revised manuscript, including how to upload and organize the files.

9) Our journal encourages inclusion of *data citations in the reference list* to directly cite datasets that were re-used and obtained from public databases. Data citations in the article text are distinct from normal bibliographical citations and should directly link to the database records from which the data can be accessed. In the main text, data citations are formatted as follows: "Data ref: Smith et al, 2001" or "Data ref: NCBI Sequence Read Archive PRJNA342805, 2017". In the Reference list, data citations must be labeled with "[DATASET]". A data reference must provide the database name, accession number/identifiers and a resolvable link to the landing page from which the data can be accessed at the end of the reference.

10) We replaced Supplementary Information with Expanded View (EV) Figures and Tables that are collapsible/expandable online. A maximum of 5 EV Figures can be typeset. EV Figures should be cited as 'Figure EV1, Figure EV2" etc... in the text and their respective legends should be included in the main text after the legends of regular figures.

- For the figures that you do NOT wish to display as Expanded View figures, they should be bundled together with their legends in a single PDF file called *Appendix*, which should start with a short Table of Content. Appendix figures should be referred to in the main text as: "Appendix Figure S1, Appendix Figure S2" etc.

- Additional Tables/Datasets should be labeled and referred to as Table EV1, Dataset EV1, etc. Legends have to be provided in a separate tab in case of .xls files. Alternatively, the legend can be supplied as a separate text file (README) and zipped together with the Table/Dataset file.

11) The paper explained: EMBO Molecular Medicine articles are accompanied by a summary of the articles to emphasize the major findings in the paper and their medical implications for the non-specialist reader. Please provide a draft summary of your article highlighting

- the medical issue you are addressing,

- the results obtained and

- their clinical impact.

This may be edited to ensure that readers understand the significance and context of the research. Please refer to any of our published articles for an example.

12) Author contributions: You will be asked to provide CRediT (Contributor Role Taxonomy) terms in the submission system. These replace a narrative author contribution section in the manuscript.

13) A Conflict of Interest statement should be provided in the main text.

14) Every published paper includes a 'Synopsis' to further enhance discoverability. Synopses are displayed on the journal webpage and are freely accessible to all readers. They include a short stand first (maximum of 300 characters, including space) as well as 2-5 one-sentences bullet points that summarizes the paper. Please write the bullet points to summarize the key NEW findings. They should be designed to be complementary to the abstract - i.e. not repeat the same text. We encourage inclusion of key acronyms and quantitative information (maximum of 30 words / bullet point). Please use the passive voice. Please attach these in a separate file or send them by email, we will incorporate them accordingly.

Please also suggest a striking image or visual abstract to illustrate your article as a PNG file 550 px wide x 300 px high.

15) Include a Reagents and Tools Table as part of the Methods section, which can be downloaded from our author guidelines.

EMBO Molecular Medicine has a "scooping protection" policy, whereby similar findings that are published by others during review or revision are not a criterion for rejection. Should you decide to submit a revised version, I do ask that you get in touch after three months if you have not completed it, to update us on the status.

*Additional important information regarding Figures

Each figure should be given in a separate file and should have the following resolution:

Graphs 800-1,200 DPI
Photos 400-800 DPI
Colour (only CMYK) 300-400 DPI"

Figures are not edited by the production team. All lettering should be the same size and style; figure panels should be indicated by capital letters (A, B, C etc). Gridlines are not allowed except for log plots. Figures should be numbered in the order of their appearance in the text with Arabic numerals. Each Figure must have a separate legend and a caption is needed for each panel.

*Additional important information regarding figures and illustrations can be found at https://media.springernature.com/original/springer-cms/rest/v1/content/27825798/data/v1

***** Reviewer's comments *****

Referee #2 (Remarks for Author):

The authors have sufficiently addressed my initial concerns with the submission. Congratulations on a well executed study and interesting analysis

Referee #3 (Remarks for Author):

The authors have addressed my comments adequately.

The authors addressed the remaining editorial issues.

2nd Jul 2026

Dear Dr. Cantaert,

We are pleased to inform you that your manuscript is accepted for publication and is now being sent to our publisher to be included in the next available issue of EMBO Molecular Medicine.

Your manuscript will be processed for publication by EMBO Press. It will be copy edited and you will receive page proofs prior to publication. Please note that you will be contacted by Springer Nature Author Services to complete licensing and payment information.

You may qualify for financial assistance for your publication charges - either via a Springer Nature fully open access agreement or an EMBO initiative. Check your eligibility: https://link.springer.com/journal/44321/how-to-publish-with-us

Should you be planning a Press Release on your article, please notify the production team via this form in order to coordinate publication and release dates: https://forms.cloud.microsoft/e/eH4A8JVP6g

If you have any questions, please do not hesitate to contact the Editorial Office. Thank you for your contribution to EMBO Molecular Medicine.

Yours sincerely,
Zeljko Durdevic

Zeljko Durdevic
Senior Editor
EMBO Molecular Medicine

>>> Please note that it is EMBO Molecular Medicine policy for the transcript of the editorial process (containing referee reports and your response letter) to be published as an online supplement to each paper. If you do NOT want this, you will need to inform the Editorial Office via email immediately. More information is available here: https://link.springer.com/partners/embo-press/editorial-policies#Peer%20review

EMBO Press Author Checklist

Corresponding Author Name: Tineke Cantaert	
Journal Submitted to: EMBO Molecular Medicine	
Manuscript Number: EMM-2026-23675-T	

USEFUL LINKS FOR COMPLETING THIS FORM
The EMBO Journal - Author Guidelines
EMBO Reports - Author Guidelines
Molecular Systems Biology - Author Guidelines
EMBO Molecular Medicine - Author Guidelines

Reporting Checklist for Life Science Articles (updated January
This checklist is adapted from Materials Design Analysis Reporting (MDAR) Checklist for Authors. MDAR establishes a minimum set of requirements in transparent reporting in the life sciences (see Statement of Task: 10.31222/osf.io/9sm4x). Please follow the journal's guidelines in preparing your
Please note that a copy of this checklist will be published alongside your article.

Abridged guidelines for figures
1. Data
The data shown in figures should satisfy the following conditions:
- the data were obtained and processed according to the field's best practice and are presented to reflect the results of the experiments in an accurate and unbiased manner.
- ideally, figure panels should include only measurements that are directly comparable to each other and obtained with the same assay.
- plots include clearly labeled error bars for independent experiments and sample sizes. Unless justified, error bars should not be shown for technical
- if n<5, the individual data points from each experiment should be plotted. Any statistical test employed should be justified.
- Source Data should be included to report the data underlying figures according to the guidelines set out in the authorship guidelines on Data

2. Captions
Each figure caption should contain the following information, for each panel where they are relevant:
- a specification of the experimental system investigated (eg cell line, species name).
- the assay(s) and method(s) used to carry out the reported observations and measurements.
- an explicit mention of the biological and chemical entity(ies) that are being measured.
- an explicit mention of the biological and chemical entity(ies) that are altered/varied/perturbed in a controlled manner.
- the exact sample size (n) for each experimental group/condition, given as a number, not a range;
- a description of the sample collection allowing the reader to understand whether the samples represent technical or biological replicates (including how many animals, litters, cultures, etc.).
- a statement of how many times the experiment shown was independently replicated in the laboratory.
- definitions of statistical methods and measures:
  - common tests, such as t-test (please specify whether paired vs. unpaired), simple χ2 tests, Wilcoxon and Mann-Whitney tests, can be unambiguously identified by name only, but more complex techniques should be described in the methods section;
  - are tests one-sided or two-sided?
  - are there adjustments for multiple comparisons?
  - exact statistical test results, e.g., P values = x but not P values < x;
  - definition of 'center values' as median or average;
  - definition of error bars as s.d. or s.e.m.

Please complete ALL of the questions below.
Select "Not Applicable" only when the requested information is not relevant for your study.

Materials

Newly Created Materials	Information included in the manuscript?	In which section is the information available? (Reagents and Tools Table, Materials and Methods, Figures, Data Availability Section)
New materials and reagents need to be available; do any restrictions apply?	Yes	Materials and Methods

Antibodies	Information included in the manuscript?	In which section is the information available? (Reagents and Tools Table, Materials and Methods, Figures, Data Availability Section)
For **antibodies** provide the following information:<br>- Commercial antibodies: RRID (if possible) or supplier name, catalogue number and or/clone number<br>- Non-commercial: RRID or citation	Yes	Materials and Methods

DNA and RNA sequences	Information included in the manuscript?	In which section is the information available? (Reagents and Tools Table, Materials and Methods, Figures, Data Availability Section)
**Short novel DNA or RNA including primers, probes**: provide the sequences.	Not Applicable	

Cell materials	Information included in the manuscript?	In which section is the information available? (Reagents and Tools Table, Materials and Methods, Figures, Data Availability Section)
**Cell lines:** Provide species information, strain. Provide accession number in repository **OR** supplier name, catalog number, clone number, and/**OR** RRID.	Yes	Materials and Methods
**Primary cultures:** Provide species, strain, sex of origin, genetic modification status.	Not Applicable	
Report if the cell lines were recently **authenticated** (e.g., by STR profiling) and tested for mycoplasma contamination.	Yes	Materials and Methods

Experimental animals	Information included in the manuscript?	In which section is the information available? (Reagents and Tools Table, Materials and Methods, Figures, Data Availability Section)
**Laboratory animals or Model organisms:** Provide species, strain, sex, age, genetic modification status. Provide accession number in repository **OR** supplier name, catalog number, clone number, **OR** RRID.	Not Applicable	
**Animal observed in or captured from the field:** Provide species, sex, and age where possible.	Not Applicable	
Please detail **housing and husbandry conditions**.	Not Applicable	

Plants and microbes	Information included in the manuscript?	In which section is the information available? (Reagents and Tools Table, Materials and Methods, Figures, Data Availability Section)
**Plants:** provide species and strain, ecotype and cultivar where relevant, unique accession number if available, and source (including location for collected wild specimens).	Not Applicable	
**Microbes:** provide species and strain, unique accession number if available, and source.	Yes	Materials and Methods

Human research participants	Information included in the manuscript?	In which section is the information available? (Reagents and Tools Table, Materials and Methods, Figures, Data Availability Section)
If collected and within the bounds of privacy constraints report on age, sex and gender or ethnicity for all study participants.	Yes	Demographic table

Core facilities	Information included in the manuscript?	In which section is the information available? (Reagents and Tools Table, Materials and Methods, Figures, Data Availability Section)
If your work benefited from core facilities, was their service mentioned in the acknowledgments section?	Not Applicable	

Design

Study protocol	Information included in the manuscript?	In which section is the information available? (Reagents and Tools Table, Materials and Methods, Figures, Data Availability Section)
If study protocol has been **pre-registered, provide DOI in the manuscript**. For clinical trials, provide the trial registration number **OR** cite DOI.	Not Applicable	
Report the **clinical trial registration number** (at ClinicalTrials.gov or equivalent), where applicable.	Not Applicable	

Laboratory protocol	Information included in the manuscript?	In which section is the information available? (Reagents and Tools Table, Materials and Methods, Figures, Data Availability Section)
Provide DOI OR other citation details if **external detailed step-by-step protocols** are available.	Yes	Materials and Methods

Experimental study design and statistics	Information included in the manuscript?	In which section is the information available? (Reagents and Tools Table, Materials and Methods, Figures, Data Availability Section)
Include a statement about **sample size** estimate even if no statistical methods were used.	Not Applicable	
Were any steps taken to minimize the effects of subjective bias when allocating animals/samples to treatment (e.g. **randomization procedure**)? If yes, have they been described?	Not Applicable	
Include a statement about **blinding** even if no blinding was done.	Not Applicable	
Describe **inclusion/exclusion criteria** if samples or animals were excluded from the analysis. Were the criteria pre-established?<br><br>If sample or data points were omitted from analysis, report if this was due to attrition or intentional exclusion and provide justification.	Not Applicable	
For every figure, are **statistical tests** justified as appropriate? Do the data meet the assumptions of the tests (e.g., normal distribution)? Describe any methods used to assess it. Is there an estimate of variation within each group of data? Is the variance similar between the groups that are being statistically compared?	Yes	Result section, Figure legend, and Materials and Methods

Sample definition and in-laboratory replication	Information included in the manuscript?	In which section is the information available? (Reagents and Tools Table, Materials and Methods, Figures, Data Availability Section)
In the figure legends: state number of times the experiment was **replicated** in laboratory.	Yes	Figure legend
In the figure legends: define whether data describe **technical or biological replicates**.	Yes	Figure legend

Ethics

Ethics	Information included in the manuscript?	In which section is the information available? (Reagents and Tools Table, Materials and Methods, Figures, Data Availability Section)
Studies involving **human participants**: State details of **authority granting ethics approval** (IRB or equivalent committee(s), provide reference number for approval.	Yes	Materials and Methods
Studies involving **human participants**: Include a statement confirming that **informed consent** was obtained from all subjects and that the experiments conformed to the principles set out in the WMA Declaration of Helsinki and the Department of Health and Human Services Belmont Report.	Yes	Materials and Methods
Studies involving **human participants: For publication of patient photos**, include a statement confirming that consent to publish was obtained.	Not Applicable	
Studies involving experimental **animals**: State details of **authority granting ethics approval** (IRB or equivalent committee(s), provide reference number for approval. Include a statement of compliance with ethical regulations.	Not Applicable	
Studies involving **specimen and field samples:** State if relevant **permits** obtained, provide details of authority approving study; if none were required, explain why.	Yes	Materials and Methods

Dual Use Research of Concern (DURC)	Information included in the manuscript?	In which section is the information available? (Reagents and Tools Table, Materials and Methods, Figures, Data Availability Section)
Could your study fall under dual use research restrictions? Please check biosecurity documents and list of **select agents and toxins** (CDC): https://www.selectagents.gov/sat/list.htm	Not Applicable	
If you used a select agent, is the security level of the lab appropriate and reported in the manuscript?	Not Applicable	
If a study is subject to dual use research of concern regulations, is the name of the **authority granting approval and reference number** for the regulatory approval provided in the manuscript?	Not Applicable	

Reporting

The MDAR framework recommends adoption of discipline-specific guidelines, established and endorsed through community initiatives. Journals have their own policy about requiring specific guidelines and recommendations to complement MDAR.

Adherence to community standards	Information included in the manuscript?	In which section is the information available? (Reagents and Tools Table, Materials and Methods, Figures, Data Availability Section)
State if relevant guidelines or checklists (e.g., **ICMJE, MIBBI, ARRIVE, PRISMA**) have been followed or provided.	Not Applicable	
For **tumor marker prognostic studies,** we recommend that you follow the **REMARK** reporting guidelines (see link list at top right). See author guidelines, under 'Reporting Guidelines'. Please confirm you have followed these guidelines.	Not Applicable	
For **phase II and III randomized controlled trials**, please refer to the **CONSORT** flow diagram (see link list at top right) and submit the CONSORT checklist (see link list at top right) with your submission. See author guidelines, under 'Reporting Guidelines'. Please confirm you have submitted this list.	Not Applicable	

Data Availability

Data availability	Information included in the manuscript?	In which section is the information available? (Reagents and Tools Table, Materials and Methods, Figures, Data Availability Section)
Have **primary datasets** been deposited according to the journal's guidelines (see 'Data Deposition' section) and the respective accession numbers provided in the Data Availability Section?	Not Applicable	
Were **human clinical and genomic datasets** deposited in a public access-controlled repository in accordance to ethical obligations to the patients and to the applicable consent agreement?	Not Applicable	
Are **computational models** that are central and integral to a study available without restrictions in a machine-readable form? Were the relevant accession numbers or links provided?	Not Applicable	
If publicly available data were reused, provide the respective **data citations in the reference list**.	Not Applicable	
