# Supplementary material for: Dengue virus envelope epitope-specific antibodies are associated with protection from severe dengue
Source: EMBO Mol Med. 2026 Aug 6;18(9):3448–73. doi: 10.1038/s44321-026-00485-7 (PMC13562538; doi:10.1038/s44321-026-00485-7)
Supplement: Supplementary file 2 [file 44321_2026_485_MOESM9_ESM.pdf]

Expanded View Figures

Figure EV1. Distribution of anti-E antibodies targeting diverse epitopes across different infection phases in the Cambodian cohort.

DENV E protein epitope-specific Abs in the plasma of dengue patients who had confirmed secondary infection with DENV2 were detected using MIA. Plasma samples were collected during (**A**) the acute phase (0–3 days after onset of fever; $n = 46$) (all $p < 0.0001$ except anti-sE Vs total sE-dimer Abs ($p = 0.0016$)), (**B**) critical phase (4–6 days after onset of fever; $n = 57$) (all $p < 0.0001$ except anti-EDIII Vs sE-dimer specific Abs ($p = 0.0243$)), (**C**) early recovery phase (7–14 days after onset of fever; $n = 65$) (all $p < 0.0001$ except anti-EDIII Vs sE-dimer specific Abs ($p = 0.0002$) and 4G2-like Abs ($p = 0.0020$)), and (**D**) the recovery phase (60 days after onset of fever; $n = 48$) (all $p < 0.0001$ except anti-EDIII Vs sE-dimer specific Abs ($p = 0.0019$) and Total sE-dimer Vs 4G2-like Abs ($p = 0.0001$)). Comparison of the Abs binding to EDIII (Blue dots), sE (green dots), total sE-dimer (orange dots), proportion of 4G2-like Abs (light green triangles), and proportion of sE-dimer specific Abs (light orange triangles). Each line represents an individual, and the data shown as MFI IgG or proportion MFI IgG (ΔMFI IgG). Paired Bonferroni's multiple comparison test was used to compare multiple groups in panels (**A**–**D**). All experiments were performed in single.

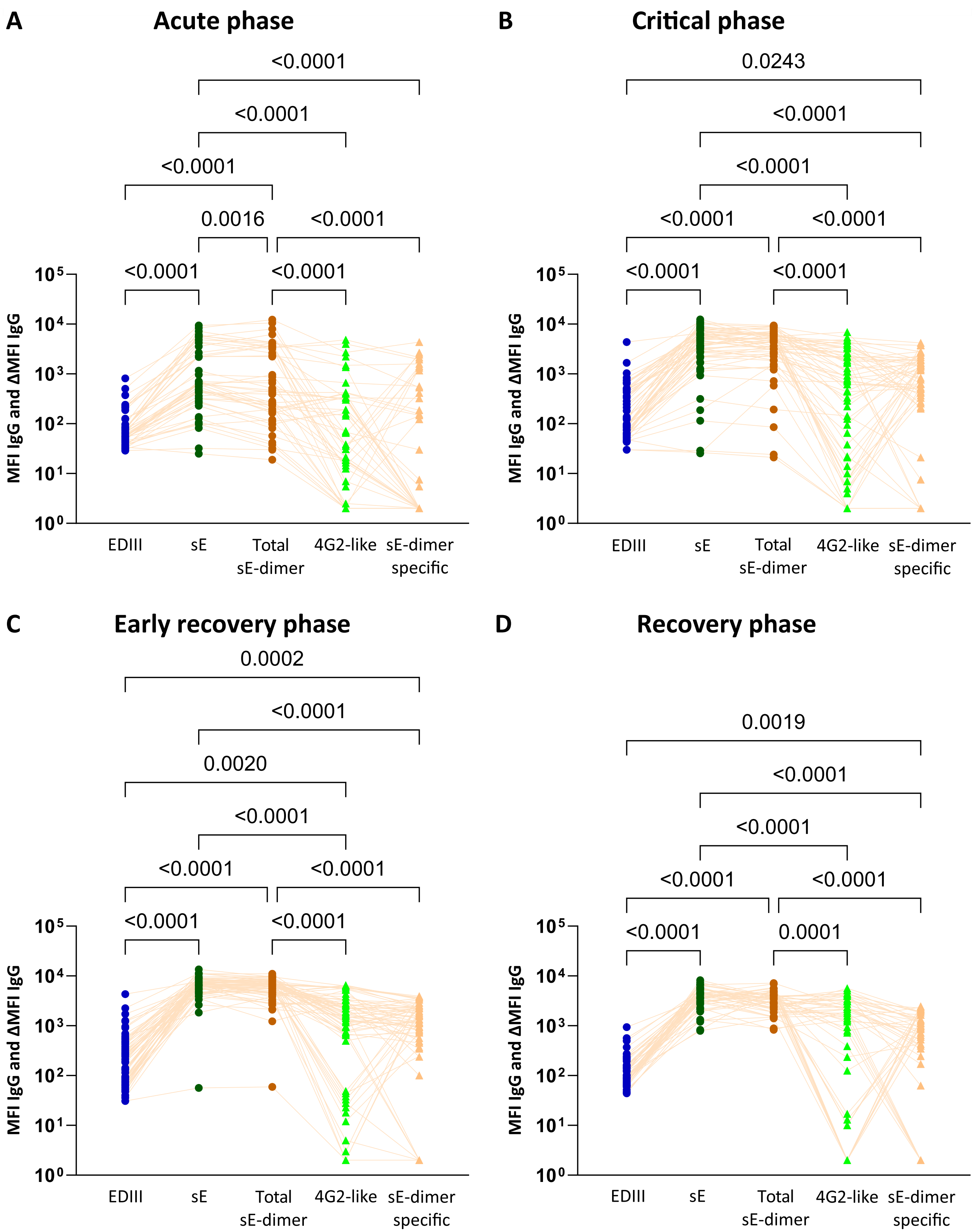
A
Acute phase
<0.0001
<0.0001
<0.0001
0.0016
<0.0001
<0.0001
<0.0001
MFI IgG and ΔMFI IgG
EDIII
sE
Total sE-dimer
4G2-like
sE-dimer specific
B
Critical phase
0.0243
<0.0001
<0.0001
<0.0001
<0.0001
<0.0001
<0.0001
C
Early recovery phase
0.0002
<0.0001
0.0020
<0.0001
<0.0001
<0.0001
<0.0001
<0.0001
D
Recovery phase
0.0019
<0.0001
<0.0001
<0.0001
<0.0001
<0.0001
0.0001

 © The Author(s)

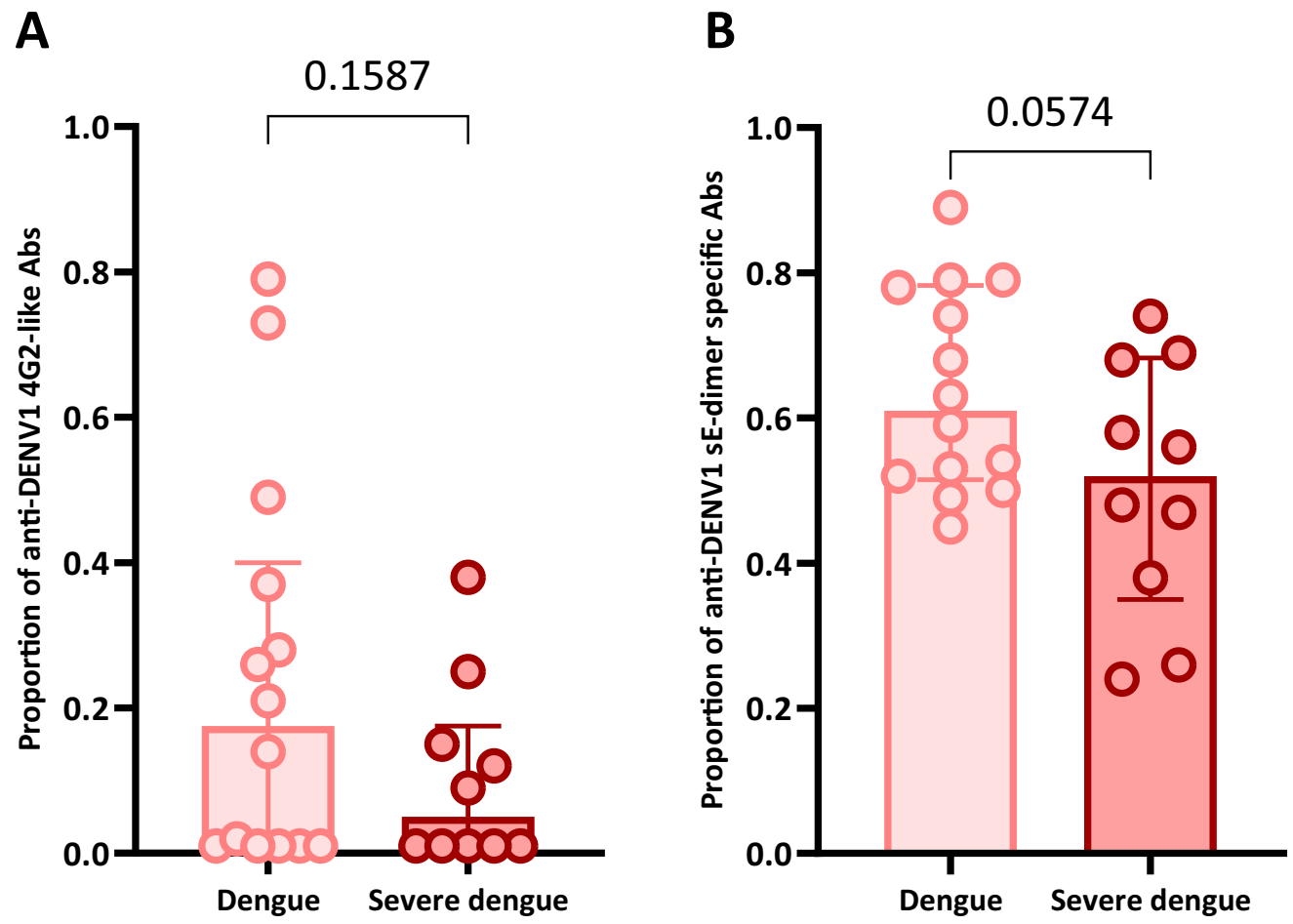

Figure EV2. Comparison of the proportions of 4G2-like and sE-dimer specific antibodies in Colombian hospitalized patients.

Plasma samples were collected from Colombian patients with confirmed secondary DENV1 infection at 2–7 days after the onset of symptoms. Anti-DENV1 antibody (Ab) subsets were measured using microsphere-based immunoassay (MIA) using DENV1-derived sE, sE-dimer and EDIII recombinant proteins. (**A**) Proportion of 4G2-like Abs ($p = 0.1587$), and (**B**) proportion of sE-dimer specific Abs ($p = 0.0574$) were compared between dengue ($n = 14$) and severe dengue ($n = 10$) cases. Each dot represents an individual; box-and-whisker plots display the median and interquartile range. Statistical comparison between groups was performed using the unpaired *T*-test.

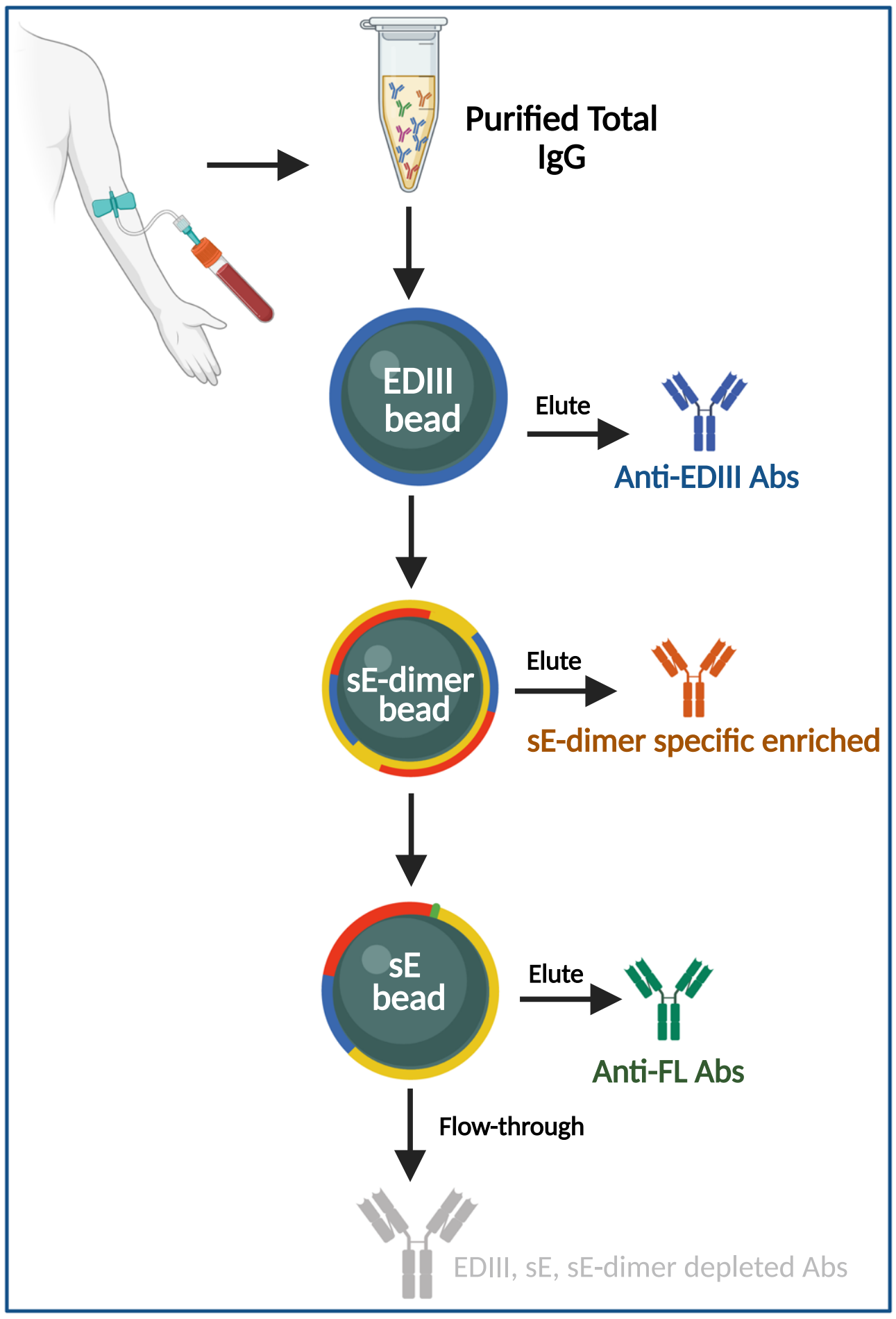

Figure EV3. Scheme of DENV E protein Epitope-specific Abs isolation from human plasma.

Total IgG was isolated from the plasma of DENV2-infected individuals and used for epitope-specific antibody purification. Site-specific biotinylated DENV2 E protein antigens (EDIII, sE, and sE-dimer) were immobilized on streptavidin-coated resin beads. The negative and positive selection were used, and purification of the isolated fraction were confirmed by MIA. Details of the protocol and validation of the isolation has been published before (Lay et al, 2025). All experiments were performed in single.

 © The Author(s)

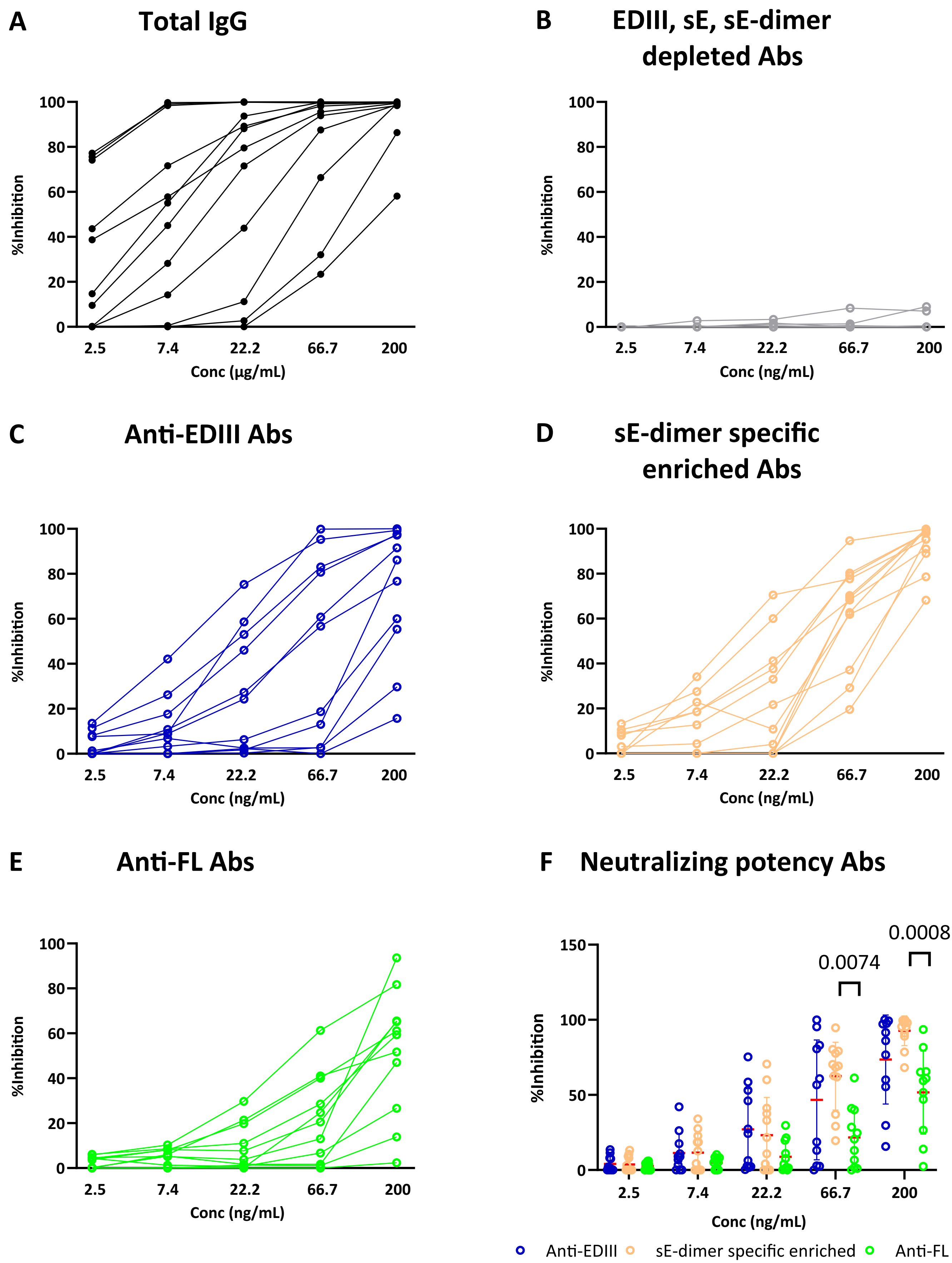
A
Total IgG
%Inhibition
Conc (µg/mL)
B
EDIII, sE, sE-dimer depleted Abs
%Inhibition
Conc (ng/mL)
C
Anti-EDIII Abs
%Inhibition
Conc (ng/mL)
D
sE-dimer specific enriched Abs
%Inhibition
Conc (ng/mL)
E
Anti-FL Abs
%Inhibition
Conc (ng/mL)
F
Neutralizing potency Abs
%Inhibition
0.0074
0.0008
Conc (ng/mL)
2.5
7.4
22.2
66.7
200
Anti-EDIII
sE-dimer specific enriched
Anti-FL

Figure EV4. Neutralizing capacity of isolated anti-DENV E protein antibody fractions.

Neutralizing activity was assessed using a flow cytometry-based assay in which Vero CCL-81 cells were infected with DENV2 in the presence or absence of serially diluted antibody (Ab) fractions. Due to the low amount of epitope-specific antibodies, experiments were performed in single. In one individual, insufficient amounts of anti-EDIII antibodies could be purified, while in another individual, insufficient amounts of anti-FL antibodies could be purified. Shown are the neutralization profiles for: (**A**) total IgG ($n = 12$), (**B**) EDIII, sE, and sE-dimer depleted Abs ($n = 12$), (**C**) anti-EDIII Abs ($n = 11$), (**D**) sE-dimer specific enriched ($n = 12$), and (**E**) anti-FL Abs ($n = 11$). (**F**) Comparison of neutralizing potency across isolated Ab fractions anti-EDIII (blue), sE-dimer-specific enriched (light orange) and anti-FL Abs (light green) at various concentrations. Percentage of inhibition was calculated following formula % inhibition = 100 – ((% infection in the presence of Abs × 100)/% infection in the absence of Abs). Each dot represents an individual, and whisker plots show the mean and standard deviation. The Dunn's multiple test was used for multiple comparisons across Ab fractions at each concentration. All experiments were performed in single.

 © The Author(s)

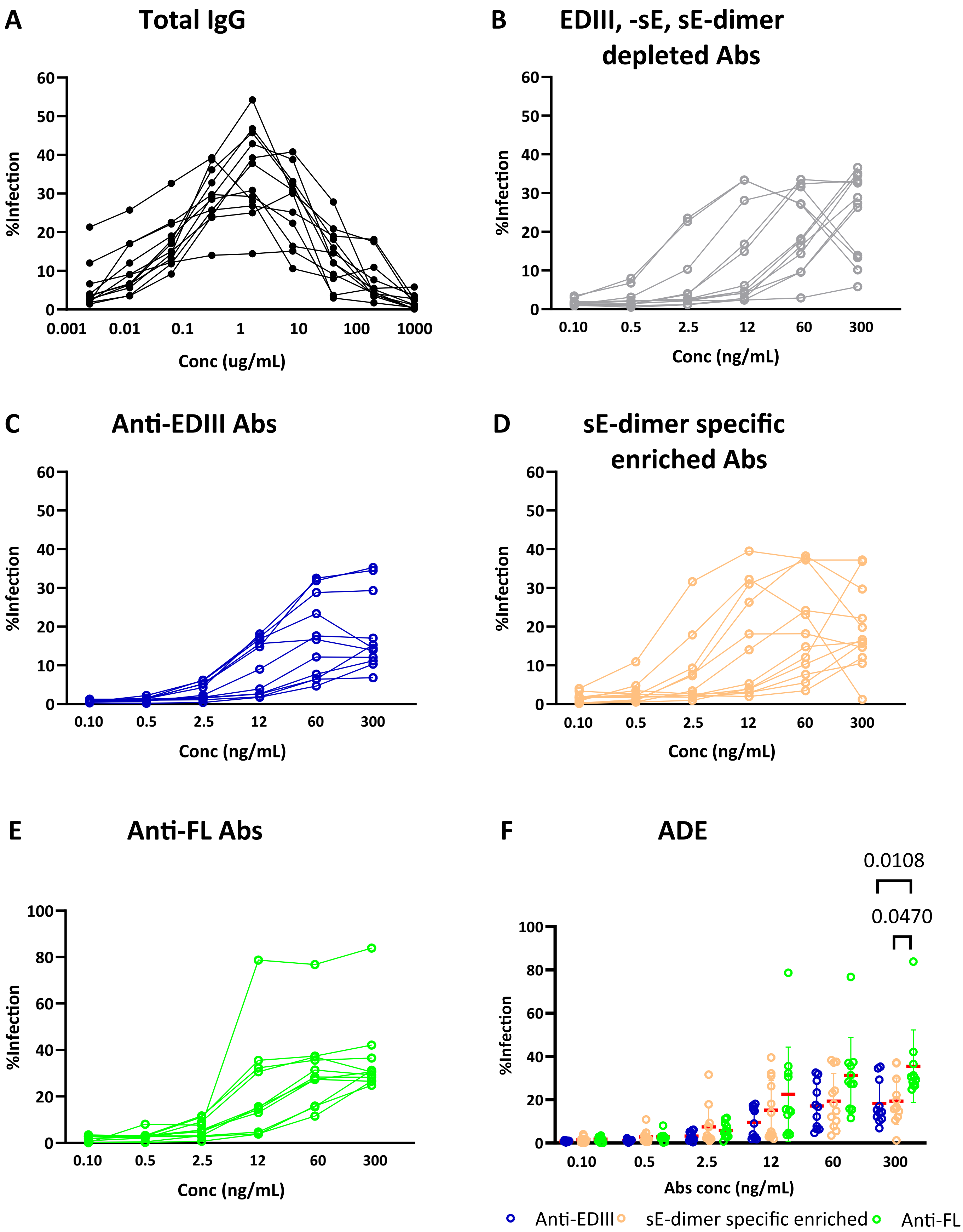
A
Total IgG
%Infection
Conc (ug/mL)
0.001
0.01
0.1
1
10
100
1000
B
EDIII, -sE, sE-dimer depleted Abs
%Infection
Conc (ng/mL)
C
Anti-EDIII Abs
%Infection
Conc (ng/mL)
D
sE-dimer specific enriched Abs
%Infection
Conc (ng/mL)
E
Anti-FL Abs
%Infection
Conc (ng/mL)
F
ADE
0.0108
0.0470
%Infection
Abs conc (ng/mL)
0.10
0.5
2.5
12
60
300
Anti-EDIII
sE-dimer specific enriched
Anti-FL

◀ **Figure EV5. Antibody-dependent enhancement (ADE) activity of isolated anti-DENV E protein antibody fractions.**

The enhancing potential of isolated antibody (Ab) fractions was assessed using an in vitro ADE assay with U937-panFcγR expressing cells. Due to the low amount of epitope-specific antibodies, experiments were performed in single. In one individual, insufficient amounts of anti-EDIII antibodies could be purified, while in another individual, insufficient amounts of anti-FL antibodies could be purified. The cells were infected with DENV2 in the absence or presence of serially diluted Ab fractions: (**A**) total IgG ($n = 12$), (**B**) EDIII, sE, sE-dimer depleted Abs ($n = 12$), (**C**) anti-EDIII Abs ($n = 11$), (**D**) sE-dimer specific enriched Abs ($n = 12$), and (**E**) anti-FL Abs ($n = 11$). (**F**) Comparison of ADE activity among anti-EDIII (blue; $n = 11$), sE-dimer-specific enriched (light orange; $n = 12$), and anti-FL (light green; $n = 11$) Ab fractions across a range of various concentrations. Each line represents the percentage of infected U937 cells induced by Ab fractions from an individual donor. The whisker plots show the mean and standard deviation. The Nonparametric Dunn's test was used for multiple comparisons between the Ab fraction at each concentration.

 © The Author(s)
