# Supplementary material for: Dengue virus envelope epitope-specific antibodies are associated with protection from severe dengue
Source: EMBO Mol Med. 2026 Aug 6;18(9):3448–73. doi: 10.1038/s44321-026-00485-7 (PMC13562538; doi:10.1038/s44321-026-00485-7)
Supplement: Supplementary file 3 [file 44321_2026_485_MOESM1_ESM.docx]

**Table EV1**.

| **Cohort 1**  **(Cambodia)** | Subclinical dengue | DF | DHF/DSS | Overall |
| --- | --- | --- | --- | --- |
|  | (N=19) | (N=25) | (N=26) | (N=70) |
| **Age (year)** | | | | |
| Mean (SD) | 10.6 (4.9) | 10.9 (3.6) | 10.4 (2.9) | 10.7 (3.7) |
| **Gender** | | | | |
| Female | 7 (37%) | 12 (48%) | 14 (54%) | 33 (47%) |
| Male | 12 (63%) | 13 (52%) | 12 (46%) | 37 (53%) |
| **Day of fever** | | | | |
| Mean (SD) | 3.5 (2.8) | 2.4 (1.0) | 3.1 (0.9) | 2.9 (1.4) |
| **RT-qPCR (Ct value)** | | | | |
| Mean (SD) | 31.0 (6.8) | 27.0 (5.5) | 31.0 (6.4) | 29.5 (6.4) |
| **No. of samples in each phase of infection** | | | | |
| Acute | 13 | 21 | 12 | 46 |
| Critical | 9 | 25 | 23 | 57 |
| Early recovery | 17 | 25 | 23 | 65 |
| Recovery | 8 | 25 | 15 | 48 |

| **Cohort 2**  **(Colombia)** | Dengue | Severe dengue | Overall |
| --- | --- | --- | --- |
|  | (N=14) | (N=10) | (N=24) |
| **Age (year)** | | | |
| Mean (SD) | 21.4 (19.8) | 25.6 (13.38) | 23.1 (17.2) |
| **Gender** | | | |
| Female | 5 (36%) | 6 (60%) | 11 (46%) |
| Male | 9 (64%) | 4 (40%) | 13 (54%) |
| **Day of fever** | | | |
| Mean (SD) | 5.1 (0.6) | 4.7 (0.6) | 4.9 (0.6) |

DF: dengue fever, DHF: dengue hemorrhagic fever, DSS: dengue shock syndrome, SD: standard deviation.

**Table EV1. Demographic and clinical characteristics of the patient cohorts**. All patients were diagnosed as confirmed DENV-infection by RT-qPCR. All patients in cohort 1 were confirmed DENV2-infected, while all patients in cohort 2 were confirmed DENV1-infected by serotype-specific PCR.
